# Redundancy in Anaerobic Digestion Microbiomes during Disturbances by the Antibiotic Monensin

**DOI:** 10.1128/AEM.02692-17

**Published:** 2018-04-16

**Authors:** Catherine M. Spirito, Sarah E. Daly, Jeffrey J. Werner, Largus T. Angenent

**Affiliations:** a Department of Biological and Environmental Engineering, Cornell University, Ithaca, New York, USA; b Chemistry Department, SUNY-Cortland, Cortland, New York, USA; c Center for Applied Geosciences, University of Tübingen, Tübingen, Germany; Centers for Disease Control and Prevention

**Keywords:** antibiotic, disturbance, redundancy, microbiome, monensin, *Bacteroidales*, OP11, anaerobic digestion, dairy manure, hindgut

## Abstract

The antibiotic monensin is fed to dairy cows to increase milk production efficiency. A fraction of this monensin is excreted into the cow manure. Previous studies have found that cow manure containing monensin can negatively impact the performance of anaerobic digesters, especially upon first introduction. Few studies have examined whether the anaerobic digester microbiome can adapt to monensin during the operating time. Here, we conducted a long-term time series study of four lab-scale anaerobic digesters fed with cow manure. We examined changes in both the microbiome composition and function of the anaerobic digesters when subjected to the dairy antibiotic monensin. In our digesters, monensin was not rapidly degraded under anaerobic conditions. The two anaerobic digesters that were subjected to manure from monensin feed-dosed cows exhibited relatively small changes in microbiome composition and function due to relatively low monensin concentrations. At higher concentrations of monensin, which we dosed directly to control manure (from dairy cows without monensin), we observed major changes in the microbiome composition and function of two anaerobic digesters. A rapid introduction of monensin to one of these anaerobic digesters led to the impairment of methane production. Conversely, more gradual additions of the same concentrations of monensin to the other anaerobic digester led to the adaptation of the anaerobic digester microbiomes to the relatively high monensin concentrations. A member of the candidate OP11 (Microgenomates) phylum arose in this anaerobic digester and appeared to be redundant with certain Bacteroidetes phylum members, which previously were dominating.

**IMPORTANCE** Monensin is a common antibiotic given to dairy cows in the United States and is partly excreted with dairy manure. An improved understanding of how monensin affects the anaerobic digester microbiome composition and function is important to prevent process failure for farm-based anaerobic digesters. This time series study demonstrates how anaerobic digester microbiomes are inert to low monensin concentrations and can adapt to relatively high monensin concentrations by redundancy in an already existing population. Therefore, our work provides further insight into the importance of microbiome redundancy in maintaining the stability of anaerobic digesters.

## INTRODUCTION

Anaerobic digestion is a biotechnology platform that is used to treat organic wastes and produce methane. It is a mature technology, which is applied around the world on farms and at municipal and industrial wastewater treatment plants. In the controlled environment of an anaerobic digester, a complex microbial food web breaks down organic wastes via hydrolysis, acidogenesis, acetogenesis, and methanogenesis to the main end product gaseous methane. The process stability relies on syntrophic interactions between microbes carrying out different components of the anaerobic digestion pathway (1). In anaerobic digestion, multiple populations are often able to carry out the same function in the digester, which leads to a flexible community structure and increased process stability via redundancy (i.e., the replacement of negatively impacted populations by others to fill their ecological niche [2]) in the microbiome (3). An increased understanding of how anaerobic digester microbiomes respond to disturbances is important from both microbial ecology and bioprocess operating perspectives.

The introduction of an inhibiting compound, such as the antibiotic monensin, can result in digester disturbance. Monensin is a monovalent carboxylic ionophore antibiotic that is given to dairy cows to increase their milk production efficiency or to treat coccidiosis, which is a parasitic infection (4). Monensin alters the fermentation pathways in the cow rumen by inhibiting the formation of precursors of methanogenesis. A previous study by Chen and Wolin (5) demonstrated that monensin favors the proliferation of propionate-producing bacteria and inhibits hydrogen-, formate-, and acetate-producing bacteria, leading to decreased methane production in the cow rumen. Similar to other ionophore antibiotics, monensin accumulates in the cell membranes of bacteria and interferes with the ion gradients needed to transport nutrients and generate the proton motive force. The affected bacterium diverts its ATP away from other essential cell processes to try to restore the normal ion gradient, and as a result, growth ceases (4). In general, Gram-negative bacteria are considered to be more resistant to monensin than Gram-positive bacteria due to the outer lipopolysaccharide layer of the Gram-negative bacteria (4). However, the division is not always clear-cut. A pure culture study by Simjee et al. (4) with three strains of Gram-positive bacteria isolated from cattle rumen (i.e., Clostridium perfringens, Enterococcus faecium, and Enterococcus faecalis) indicated that these bacteria can develop monensin resistance through altered cell membrane characteristics, such as thickening of the extracellular polysaccharides (glycocalyx) layer or the cell wall. Similarly, Rychlik and Russell (6) observed the adaptation to monensin by a strain of the Gram-positive amino acid-fermenting bacterium Clostridium aminophilum. Conversely, some Gram-negative bacteria, such as certain strains of Prevotella bryantii (7) and Prevotella ruminicola (8), are sensitive to monensin and require an adaptation period.

Previous studies and on-farm experiences have shown a decrease in methane production upon the first introduction of monensin or monensin-laden manure to anaerobic digesters (9–12). These studies indicated that monensin indirectly inhibits methanogenesis by depleting the precursor acetate. However, a 6-month study by Varel and Hashimoto (13) indicated that an anaerobic digester is capable of adapting to monensin during the operating period. In that study, only the concentration of monensin in the cow feed was reported, whereas the concentration of monensin in the adapted digester was not reported. A lack of long-term studies in this area has limited the understanding of how to prevent possible digester failure or decreased performance. Some studies have been conducted on the effects of other antibiotics on the anaerobic microbiome, including the effects of tylosin (14–16), azithromycin, chloramphenicol, and kanamycin (17). One study used 454 pyrosequencing of the 16S rRNA gene to look at the effect of monensin on microbial populations in the cow rumen (18). However, few studies exist that examine how the anaerobic digester microbiome is affected by or adapts to the antibiotic monensin.

Here, a long-term time series bioreactor study (383 days) was carried out to investigate the effect of disturbances (i.e., monensin addition) on the stability and performance of the anaerobic digester microbiome. Four, lab-scale (4.5 liters), continuously stirred anaerobic digesters were fed manure from cows feed-dosed with monensin or from control cows. The four anaerobic digesters were exposed to different disturbance regimes by varying the monensin concentrations in the digester feed, either by the addition of manure from monensin feed-dosed cows or via manure from control cows with monensin directly added. Illumina 16S rRNA gene sequencing was utilized to study the changes in the microbiome composition in the anaerobic digesters over time, as well as in the manure and hindgut samples from the study cows.

## RESULTS

### Concentrations of monensin in manure from monensin feed-dosed dairy cows were relatively low compared to those from beef cattle.

Three dairy cows in this study received a diet with monensin (via a corn grain top dressing mixed with monensin), while four dairy cows received a diet with no monensin. For the monensin feed-dosed cows, the monensin concentration in the top dressing was increased every 2 weeks for a period of 2 months, according to the protocol recommended by the manufacturer (Elanco Animal Health, Indianapolis, IN) to introduce monensin to dairy cow diets. The manure from the two groups of dairy cows was collected at the end of each monensin dose period to obtain five batches of manure: M0, M200, M300, M400, and M500, corresponding to the 0, 194, 320, 432, and 546 mg · day^−1^ monensin dosing rates, respectively (M0 was collected from the control cows, while M200 to M500 were collected from the monensin feed-dosed cows). The monensin concentrations in the wet manure collected from the monensin feed-dosed cows in this study ranged from 0.239 ± 0.009 mg · liter^−1^ at the lowest monensin dosing rate of 194 mg · day^−1^ to 0.543 ± 0.011 mg · liter^−1^ at the highest monensin dosing rate of 546 mg · day^−1^ (see Table S1 in the supplemental material). We calculated a monensin excretion rate of 13% (*R*^2^ = 0.82) on the basis of the linear relationship that was observed between the measured monensin concentrations in the consumed feed and that in the manure (on a dry matter basis in mg · kg^−1^ in Fig. S1). In a prior study with steers (beef cattle) fed with monensin at 330 mg · day^−1^ per cow, 40 to 50% of the dosed monensin was excreted in the manure (19), which is considerably higher than in our study. No other study was found in the literature that reported monensin excretion rates for dairy cows, and we were not able to calculate such rates from the reported monensin concentrations in lagoon samples from dairy farms (20). Potentially, the difference in monensin excretion rates for dairy cows versus beef cattle could be partially due to differences in the diets and the rates of feed passage through the animals. In addition, we calculated the monensin dosing rates to the dairy cows on the basis of the monensin concentrations that we measured in the top dressing. This may not reflect the actual rate of monensin consumption by the dairy cows, since the cows may not have consumed all the top dressing they were presented with. Regardless, the monensin concentration in the manure from the monensin feed-dosed cows was relatively low.

### Chemical and microbial compositions of manure from monensin feed-dosed cows were different from those of manure from control cows.

The manure batches that we collected from the monensin feed-dosed cows and the control cows were diluted with various amounts of tap water prior to feeding to the anaerobic digesters to achieve the targeted organic loading rate (Table S1). We observed some differences in the physical and chemical properties of the manure from the monensin feed-dosed cows versus the control cows, which persisted after the manure was diluted (Table S1) and which, thus, could have affected the digester performance. The total alkalinity and total ammonium concentrations, as well as the pH, were significantly higher (*P* < 0.05) and the total volatile fatty acid (tVFA) concentrations were significantly lower (*P* < 0.05) for the majority of manure from the feed-dosed cows versus manure from the control cows (Table S1). The total alkalinity, total ammonium concentration, and pH generally increased with monensin dosing level (Table S1). However, the manure from the monensin feed-dosed cows was collected at later time points than the manure from the control cows, which was only collected at a single time point. Therefore, natural changes in the cow metabolism during our experimental period may also have contributed to the observed differences in the manures. Bomb calorimetry analyses revealed no significant differences in the gross energies of the manures (*P* = 0.296) (Table S1). No clear patterns emerged in the acetate-to-propionate ratios in the manures (Table S1).

We analyzed the 16S rRNA gene sequences from the combined manure substrates that were fed to the anaerobic digesters (M0, M200, M300, M400, and M500). The dominant taxon families that we observed in the manure substrates are reported in the supplemental material (see Table S2). The principal-coordinate analysis (PCoA; unweighted UniFrac) of the manure substrates (M0 to M500) showed a gradient with increasing monensin application, though the small sample size should be noted (see Fig. S2B). In addition to the combined manure substrates, which we fed to the anaerobic digesters, we also collected and characterized via Illumina 16S rRNA gene sequencing the microbiomes of hindgut samples collected from the study cows. A PCoA of unweighted UniFrac distances between cow hindgut samples revealed clustering of the monensin feed-dosed cow samples versus the control cow hindgut samples (the monensin feed-dosed cow samples are all in the left half of Fig. S2A). However, this clustering could not be exclusively attributed to the monensin dosing rates, because the cow hindgut samples clustered strongly on the basis of the individual cows they came from. Importantly, the samples still clustered within the individual cows whether they were taken 2 weeks before or during monensin dosing for each of the monensin feed-dosed cows (Fig. S2A). To determine whether monensin contributed to changes in the cow manure microbiomes, we would need to repeat the experiment on a larger scale. The combined results of the hindgut and manure sequencing work showed that the microbiomes in the manure from the monensin feed-dosed cows were different from those in the manure from the control cows, but our study design was too small to attribute monensin to this observed difference.

### After the first 200 days without monensin, the four identical anaerobic digesters had replicable microbiomes.

**FIG 1:**
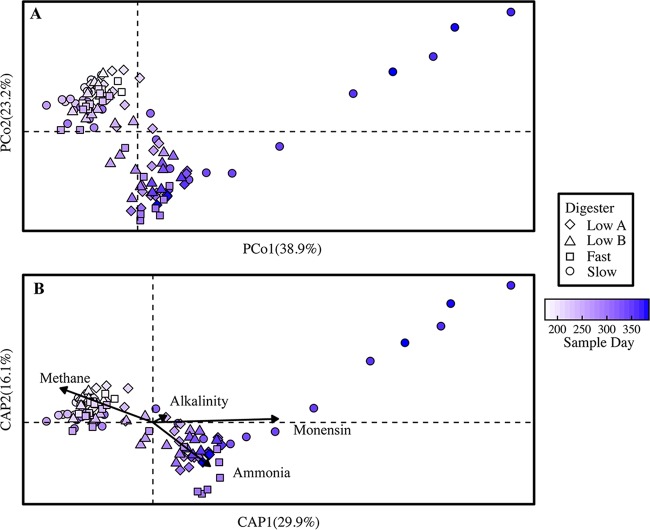
β-Diversity of all anaerobic digester microbiome samples from day 175 to the end of the operating period. (A) Principal-coordinate analysis (PCoA) plot based on weighted UniFrac distance. (B) Distance-based redundancy analysis (db-RDA) plot showing the four measured parameters that best explained the variation observed in the PCoA plot. Methane, methane yield; alkalinity, bicarbonate alkalinity concentration; ammonia, total ammonium concentration; monensin, monensin concentration in the substrate. In both the PCoA and db-RDA plots, the points are colored on a gradient scale representing the duration of the operating period in days.

The four anaerobic digesters in our study were all initially operated identically and fed with the control (M0) manure—without monensin—for a period of 202 days (startup and period 1 [P1]). These periods served to acclimate the inoculum to dairy manure. During this time, each of the anaerobic digester microbiomes exhibited a similar evolution from the inoculum (day 0) community (see Table S3) toward a community that was dominated by the phyla Bacteroidetes (61.5% ± 2.7%), the WWE1 candidate division (12.4% ± 1.8%), and Firmicutes (10.8% ± 0.4%) (on day 201 in Fig. S3A to D). The richness and unevenness (α diversity) of the anaerobic digester communities changed similarly for all four anaerobic digesters between the startup period and P1 (see Fig. S4 and S5). We observed a considerable decrease in the average number of observed species (richness) between the startup period and P1 (Fig. S4A to D), while the Gini coefficient (unevenness) increased between these periods (Fig. S5A to D). We did not find statistical differences (*P* > 0.05) between the microbiomes of the four anaerobic digesters in terms of the richness and unevenness measurements during the startup period and P1. The mean weighted UniFrac distance (measurement of β diversity) between each set of reactor samples for these periods was not significantly different (*P* > 0.05, based on the Tukey honestly significant difference [HSD] model for comparing multiple means by pairwise comparisons), indicating similar degrees of divergence of the microbiomes during the premonensin periods (Fig. 1A). This replicability between anaerobic digesters that were operated identically with a complex substrate is in agreement with other previous studies (21, 22).

### Monensin did not degrade to a large extent in the anaerobic digesters.

Following the startup and P1, the four anaerobic digesters were subjected to different monensin dosing strategies throughout periods 2 to 4 (P2, P3, and P4) (Table 1). The names of the digesters were chosen to reflect whether they received relatively low concentrations of monensin (i.e., the low A and low B anaerobic digesters) or relatively high concentrations of monensin that were introduced to the digesters either quickly (i.e., the fast anaerobic digester) or more gradually (i.e., the slow anaerobic digester). The low A and low B anaerobic digesters were fed manure from monensin feed-dosed cows from day 203 (start of P2) throughout P4 (M200 to M500). The monensin concentrations in the manure that was used as a substrate for the low A and low B anaerobic digesters were relatively low at concentrations of between 0.09 and 0.19 mg · liter^−1^ after dilution (Table 1). Low A and low B anaerobic digesters were operated identically to each other, except that the final manure batch (M500), which contained the highest concentration of excreted monensin, was introduced earlier into the low A anaerobic digester than the low B anaerobic digester (day 245 versus day 267, respectively) (Table 1). The fast and slow anaerobic digesters received manure from the control cows (M0), to which a 0.1-ml volume of a prepared monensin solution (monensin reference standard from Elanco Animal Health, Greenfield, IN, which was dissolved in methanol to achieve the targeted monensin concentration) was directly added (direct-dosed manure). The monensin concentrations for the direct-dosed manure, which was used as a substrate for the fast and slow anaerobic digesters during certain periods, were between 1 and 5 mg · liter^−1^ (Table 1). These were considerably higher than the monensin concentrations in the manure from the monensin feed-dosed cows which was fed to the low A and B anaerobic digesters. The fast anaerobic digester was fed direct-dosed manure from day 203 (P2) until the anaerobic digester was shut down on the final day of P3 (Table 1). The slow anaerobic digester initially acted as a control anaerobic digester and was fed control cow manure until day 306 (P3). After this, it was fed direct-dosed manure until the end of P4 (Table 1).

**TABLE 1:** Monensin concentrations in the influents and the effluents of the anaerobic digesters

Period	Days	Low A			Low B^*a*^			Fast^*b*^			Slow		
		Manure^*c*^	Monensin concn (mg · liter^−1^)^*d*^		Manure^*c*^	Monensin concn (mg · liter^−1^)^*d*^		Manure^*c*^	Monensin concn (mg · liter^−1^)^*d*^		Manure^*c*^	Monensin concn (mg · liter^−1^)^*d*^	
			Influent^*e*^	Effluent (day)		Influent^*e*^	Effluent (day)		Influent^*e*^	Effluent (day)		Influent^*e*^	Effluent (day)
Startup	1–114	M0	0	NA^*f*^	M0	0	NA	M0	0	NA	M0	0	NA
P1	115–202	M0	0	ND^*g*^ (201)	M0	0	ND (201)	M0	0	ND (201)	M0	0	ND (201)
P2	203–216	M200	0.09	NA	M200	0.09	NA	M0+	0.09	NA	M0	0	NA
	217–230	M300	0.12	NA	M300	0.12	NA	M0+	0.12	NA	M0	0	NA
	231–244	M400	0.15	ND (231)	M400	0.15	ND (231)	M0+	0.15	ND (231)	M0	0	ND (231)
	245–264	M500	0.19	NA	M400	0.15	NA	M0+	0.19	NA	M0	0	NA
P3	265–280	M500	0.19	0.12 (267)	M500	0.19	ND (267)	M0++	1	0.11 (267)	M0	0	ND (267)
	281–306	M500	0.19	0.14 (301)	M500	0.19	0.12 (301)	M0++	5	2.29 (301)	M0	0	ND (301)
		M500	0.19	NA	M500	0.19	NA	M0++	5	2.73 (306)	M0	0	NA
P4	307–320	M500	0.19	NA	M500	0.19	NA	Stopped			M0++	1	NA
	321–338	M500	0.19	0.11 (331)	M500	0.19	0.11 (331)	Stopped			M0++	2	0.81 (331)
	339–352	M500	0.19	NA	M500	0.19	NA	Stopped			M0++	3	NA
	353–366	M500	0.19	0.13 (365)	M500	0.19	0.13 (355)	Stopped			M0++	4	1.80 (365)
	367–383	M500	0.19	0.14 (383)	Stopped			Stopped			M0++	5	2.55 (383)

a M500 manure batch was started on day 267 and discontinued on day 355 of the operating period.

b Discontinued on day 306 of the operating period.

c M200, M300, M400, and M500 refer to the four batches of manure from monensin feed-dosed cows (corresponding to the 194, 320, 432, and 546 mg · d^−1^ monensin dosing rates, respectively). M0+ or M0++ indicates that monensin was added directly to control cow (M0) manure.

d Detection limit was 0.10 mg · liter^−1^.

e Values are based on dilution (manure was diluted before feeding).

f NA, measurement not available.

g ND, monensin not detected.

Monensin was not rapidly degraded in the anaerobic environment of the digesters (Table 1). At the relatively low concentration of monensin in the influent, which was 0.19 mg · liter^−1^ for low A and low B during the periods with the manure substrate M500 (day 245 to 383 and day 267 to 355, respectively), considerable proportions (∼74% and ∼68%, respectively) remained in the effluents at the end of these periods (Table 1). Similarly, at the relatively high concentration of monensin in the fast and slow anaerobic digesters, which we dosed with up to 5 mg · liter^−1^ monensin (between days 281 and 306 and days 367 and 383, respectively), ∼54% and ∼51%, respectively, remained in the effluents (Table 1). This result builds upon a previous shorter-term study by Varel et al. (23) in which mesophilic laboratory anaerobic digesters were fed with manure from cattle dosed with monensin. After a period of 28 days, the concentration of monensin in the digesters in that study had decreased by only 8%.

### Methane yield decrease in digesters fed manure from monensin feed-dosed cows could not be attributed to a direct inhibition of acetogenesis and methanogenesis.

**FIG 2:**
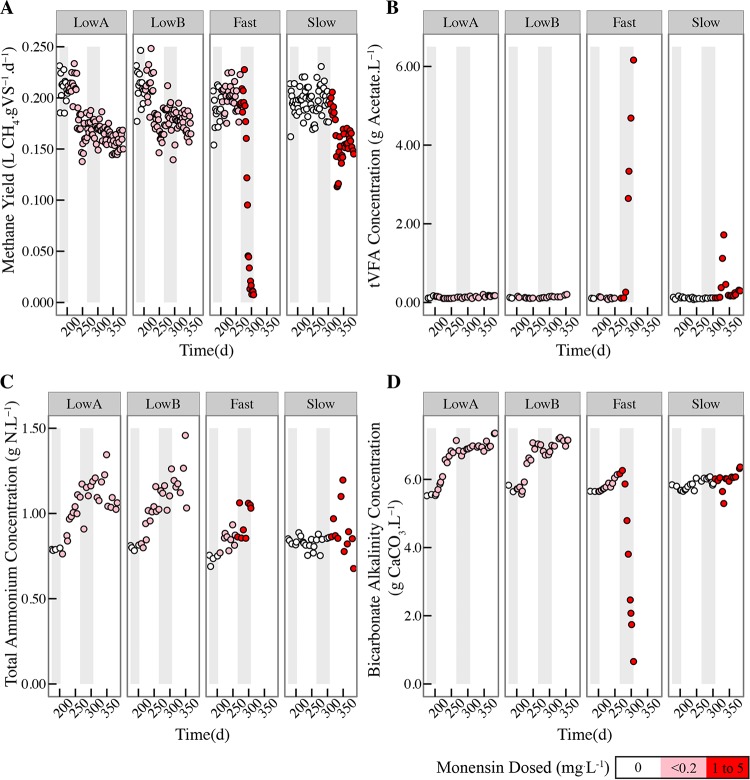
Performance parameters for the four anaerobic digesters from day 175 to the end of the operating period for specific methane yields (A), tVFA concentrations (B), total ammonium concentrations (C), and bicarbonate alkalinity concentrations (D). Points are colored on a color scale corresponding to the concentration of monensin dosed to the anaerobic digester at that time point. The different periods are marked with gray and white shading from left to right during the operating period: the first gray shading identifies P1; the first white shading identifies P2; the second gray bar identifies P3, and the second white area identifies P4 (see Table S4 in the supplemental material). The fast anaerobic digester was stopped at the end of P3 due to a loss of stability.

The low A and low B anaerobic digesters experienced decreased methane yields when they were fed the manure from the monensin feed-dosed cows during P2 and P3 in our study (Fig. 2A). Compared to the average methane yield of the slow digester in these periods (which was receiving control manure and no monensin in P2 and P3), the average methane yields were 3.2% and 1.9% lower during P2 and 14.7% and 9.9% lower during P3 for the low A and low B anaerobic digesters, respectively (Fig. 2A; Table S4). These differences were not significant for P2 (*P* = 0.581), while they were for P3 (*P* < 0.001). The monensin concentration in the manure substrate from the monensin feed-dosed cows never exceeded 0.2 mg · liter^−1^ (Table 1). A previous study by Thaveesri et al. (9) also showed that monensin at a similar low concentration of 0.1 mg · liter^−1^ decreased the performance of an upflow anaerobic sludge blanket (UASB) anaerobic digester fed with synthetic wastewater (∼20% reduction in methane yield). In our study during P2 and P3, the tVFA concentrations in the low A and low B anaerobic digesters remained low, reaching an average of 128 mg acetate (Ac) · liter^−1^ in the low A anaerobic digester and 122 mg Ac · liter^−1^ in the low B digester during P3 (see Table S5), with propionate and *n*-butyrate concentrations remaining below detection (see Table S6). Thus, it seems unlikely that that the lower methane yields were due to a direct inhibition of acetogenesis and methanogenesis by the relatively low monensin concentrations that were present in the manure from the monensin feed-dosed cows. If this had occurred, we would have anticipated a corresponding build-up of propionate and *n*-butyrate (inhibition of acetogenesis) or acetate (inhibition of methanogenesis), as has been described in other papers on anaerobic digester inhibition (1). Therefore, the introduction of monensin in the manure from the monensin feed-dosed cows into the digester did not present itself as a disturbance, and an indirect effect for the observed changes must be taken into consideration, such as a change in the manure substrate.

As noted previously, the chemical compositions of the manure from the monensin feed-dosed cows differed from those of the manure collected from the control cows (Table S1). This may have partially contributed to the decreased methane yields observed in the low A and low B digesters. Previous research has shown that the chemical quality of the substrate has an overarching effect on the anaerobic digester microbiome (24, 25). Even though the gross energy of the manure from monensin feed-dosed cows was not significantly different from that of the control manure (with bomb calorimetry analysis), we observed lower tVFA concentrations in the manure from monensin feed-dosed cows (Table S1). In addition, the concentrations of total alkalinity, total ammonium, and tVFAs, as well as the pH, had changed in the manure from monensin feed-dosed cows (Table S1). As a result, in the low A and low B anaerobic digesters, the total ammonium concentrations increased (Fig. 2C; Table S5). Furthermore, the bicarbonate alkalinity concentrations were higher for the low A and low B anaerobic digesters after the switch to the manure from monensin feed-dosed cows (Fig. 2D). This was anticipated, because ammonia ions contribute to bicarbonate alkalinity (26).

### Rapid introduction of higher monensin concentrations in the direct-dosed manure had a severe effect on methane yields.

The fast anaerobic digester received manure from the control cows to which monensin was directly dosed (direct-dosed manure). During P3, the monensin concentration in the manure substrate was rapidly increased from 0.19 to 1 mg · liter^−1^ on day 265 and then from 1 to 5 mg · liter^−1^ on day 281 (Table 1). Following the increase in monensin concentration to 5 mg · liter^−1^ on day 281, we observed a rapid decrease in methane yield and an accumulation of tVFAs in the fast anaerobic digester (total volatile fatty acids measured by the distillation method were >2 g Ac · liter^−1^ on day 291 and reached a maximum of 6.2 g Ac · liter^−1^ on day 306) (Fig. 2A and B). Gas chromatography measurements of individual volatile fatty acids indicated that the acetate concentrations increased to 38.6 mM (2.3 g · liter^−1^) on day 307 (from 0.62 mM [0.04 g · liter^−1^] on day 243) and that the propionate and *n*-butyrate concentrations increased to 9.8 mM (0.72 g · liter^−1^) and 2.8 mM (0.24 g · liter^−1^), respectively, on day 307 (from below detection on day 285 in Table S6). These measurements point toward the inhibition of both acetogenic and methanogenic populations in the fast anaerobic digester by high concentrations of monensin and a true disturbance to the microbiota. The accumulation of VFAs resulted in a drop of the pH level to 5.81 on day 306 (data not shown) and a further decrease in the methane yield to only ∼0.01 liters CH_4_ · g volatile solids (VS)^−1^ · day^−1^ was observed after which the operating period for the fast anaerobic digester was terminated due to unstable conditions (Fig. 2A).

The monensin concentrations increased more gradually in the direct-dosed manure fed to the slow anaerobic digester than in that fed to the fast anaerobic digester (Table 1). In the period (P3) prior to the addition of monensin to the slow anaerobic digester, the average methane yield in this digester was 0.20 ± 0.01 liters CH_4_ · g VS^−1^ · day^−1^ (Table S4). During P4, monensin was dosed to the slow anaerobic digester starting at 1 mg · liter^−1^ and incrementally increased by 1 mg · liter^−1^ approximately every 2 weeks until reaching 5 mg · liter^−1^ (Table 1). During this period, the methane yield initially declined but then began to recover (Fig. 2A), resulting in an average methane yield of 0.16 ± 0.02 liters CH_4_ · g VS^−1^ · day^−1^ for P4 (Table S4). We observed only temporary increases in the tVFA and individual volatile fatty acid (iVFA) concentrations in the slow anaerobic digester when the drop in the methane yield was most severe (Fig. 2A and B; Table S6). As mentioned earlier, monensin did not rapidly degrade in the anaerobic digesters, resulting in similar concentrations in both the slow and fast anaerobic digesters (Table 1). Therefore, the main difference between the fast and slow anaerobic digesters was the rate at which the concentration of monensin was increased from 1 to 5 mg · liter^−1^ (Table 1). While the fast anaerobic digester failed, the slow anaerobic digester partially recovered its performance ∼40 days after the first introduction of the monensin antibiotic, as indicated by the methane yield data (Fig. 2A).

### Higher monensin concentrations in the direct-dosed manure shifted specific OTUs in fast and slow anaerobic digesters.

**FIG 3:**
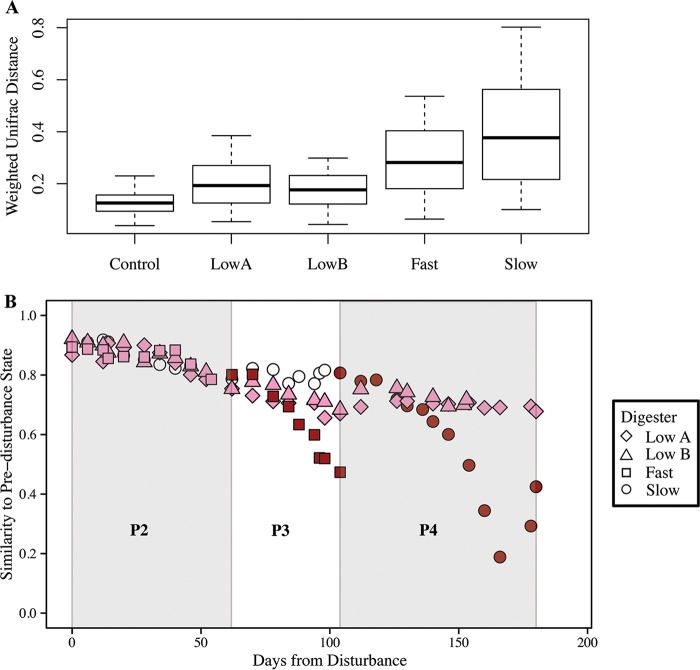
β-Diversity of anaerobic digester microbiome samples during different periods. (A) Boxplot of weighted UniFrac distances within microbiomes from a single anaerobic digester throughout a similar operating period. Control anaerobic digester boxplot corresponds to days 203 to 306, when the slow anaerobic digester was fed only control cow manure without monensin addition; the low A anaerobic digester boxplot corresponds to days 203 to 383; the low B anaerobic digester boxplot corresponds to days 203 to 355; the fast anaerobic digester boxplot only includes samples during which the anaerobic digester was subjected to high concentrations of monensin (i.e., days 265 to 306); and the slow anaerobic digester boxplot corresponds to days 306 to 383, when the anaerobic digester was subjected to high concentrations of monensin. (B) Similarity of samples postdisturbance compared to three samples prior to disturbance (prior to P2 on days 175, 189, and 201) based on the weighted UniFrac distance metric. Similarity was calculated as one minus the average weighted UniFrac distance between the sample day and the three sample days prior to disturbance (prior to P2). Colors represent the monensin concentration gradient: dark red, high monensin concentration (1 to 5 mg · liter^−1^); pink, low monensin concentration (<1 mg · liter^−1^); white, no monensin in substrate. Periods (P2 to P4) are represented by gray and white shading.

**FIG 4:**
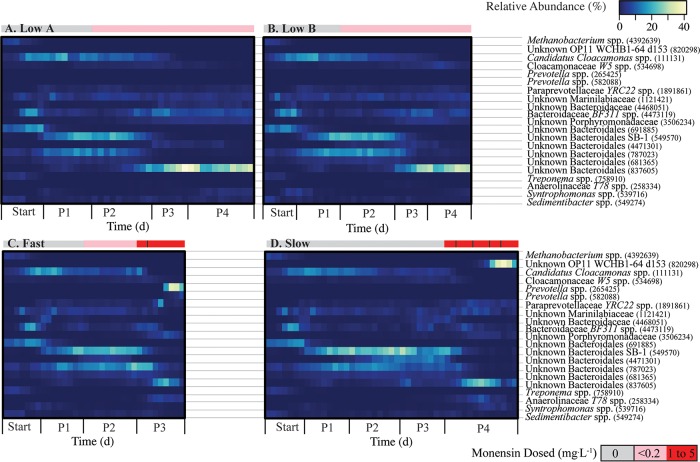
Heatmaps representing relative abundance of major OTUs in anaerobic digester samples during the entire sampling period for the low A anaerobic digester (A), low B anaerobic digester (B), fast anaerobic digester (C), and slow anaerobic digester (D). OTUs represented here reached at least 5% relative abundance in any one anaerobic digester sample. Lowest-level taxonomic data, as well as the OTU ID number, are provided. The sidebar color scale represents the monensin dosing rate to the anaerobic digester. For the fast digester (panel C), the black line in the red bar indicates when the monensin dose was directly increased from 1 to 5 mg · liter^−1^. For the slow digester (panel D), the black lines in the red bar indicate when the monensin dose was increased (from 1 to 2 mg · liter^−1^, from 2 to 3 mg · liter^−1^, from 3 to 4 mg · liter^−1^, and from 4 to 5 mg · liter^−1^).

**FIG 5:**
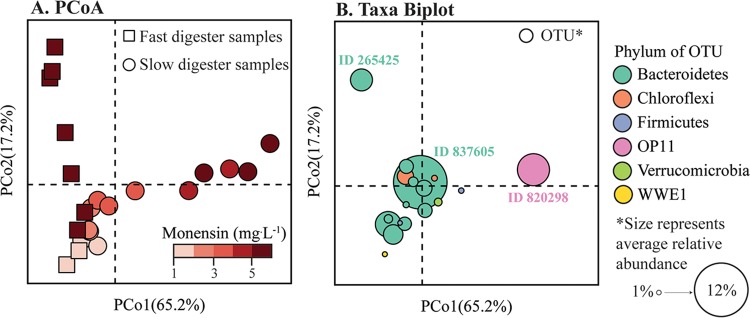
β-Diversity of the fast and slow anaerobic digester microbiome samples during high monensin periods (≥1 mg · liter^−1^; day 265 to 307 for fast anaerobic digester, and day 307 to 383 for slow anaerobic digester). (A) Principal-coordinate analysis (PCoA) based on weighted UniFrac distance. Time points are colored in a gradient scale corresponding to monensin concentration in the substrate, as indicated by the color sidebar. (B) Taxon biplot showing the most abundant OTUs in these samples (OTUs that reached an average relative abundance of ≥1% across the time period shown). OTUs are shown nearest to the samples that they are most abundant in. Sample time points are not shown as they are displayed in the left-hand PCoA. Point size represents average relative abundance of that OTU across all samples (minimum average relative abundance is 1.0%, maximum average relative abundance is 12.0%). Points are colored by phylum-level taxonomy. Three OTUs discussed in the text are highlighted. OTU 820298, OP11 OTU; OTU 265425, Prevotella OTU; OTU 837605, Bacteroidales OTU.

Initially, both the fast and slow anaerobic digesters exhibited considerable levels of resistance to the 1 mg · liter^−1^ of monensin that was added to the control manure substrate. The microbial communities of these digesters remained relatively similar to their predisturbance states for this concentration of monensin (Fig. 3B, first two data points in P3 and P4, respectively; Fig. 4C and D). Even though this monensin concentration was higher than our observed concentrations in the dairy cow manure, it is similar to monensin concentrations that were previously found to be inhibitory in anaerobic digesters (27, 28). After increasing the concentration to 5 mg · liter^−1^, the PCoA and taxon biplot (showing only the fast and slow anaerobic digester samples) clearly reveal that the microbiomes of these anaerobic digesters diverged under such relatively high concentrations of monensin (Fig. 5A and B). Two separate previously rare operational taxonomic units (OTUs) rapidly rose to high levels of relative abundance for the fast and slow anaerobic digesters during the disturbed conditions. In the next paragraph, we will discuss the OTU (a member of the genus Prevotella) that increased in the fast anaerobic digester. The OTU (a member of the OP11 phylum) that increased in the slow anaerobic digester will be discussed in a later section.

For the fast anaerobic digester, an OTU (identifier [ID] 265425) belonging to the genus Prevotella (order Bacteroidales in the phylum Bacteroidetes) rapidly increased in abundance, from not detectable on day 257 (P2, prior to high concentrations of monensin) to a maximum of 35.1% on day 299 (P3, high monensin) (Fig. 4C), while other Bacteroidales members decreased in abundance. This OTU was found at very low abundance levels in the biomass samples from the other anaerobic digesters (Fig. 4A, B, and D). The taxon biplot identified this OTU (ID 265425) as the driver that can explain the dynamics of the microbiome (Fig. 5A and B). Other Prevotella spp. OTUs also increased in the fast anaerobic digester, but to a lesser extent (data not shown). Previous studies have found various levels of resistance to monensin within the genus of Prevotella, with some members of this genus requiring a period of time to adapt to this antibiotic (4, 7, 8, 29). In the study by Callaway and Russell (7), strains of Prevotella bryantii were initially sensitive to monensin at concentrations greater than 0.7 mg · liter^−1^. However, upon repeated exposure to monensin, certain strains were resistant to monensin at concentrations as high as 7 mg · liter^−1^. The Prevotella spp. OTU relative abundance decreased in the final 2 days of the study (Fig. 4C), which suggests that monensin concentrations had reached an inhibitory threshold for this OTU. Because the fast anaerobic digester did not recover its function during the rise of the Prevotella spp. OTU, we cannot conclude that this OTU was redundant with the other Bacteroidales members that had previously dominated the anaerobic digester. Different members of Prevotella spp. also increased in the slow anaerobic digester upon monensin addition, though to a much lower extent than in the fast anaerobic digester.

### Microbiome redundancy prevented anaerobic digester failure after a disturbance by high concentrations of monensin.

When monensin was initially introduced into the slow anaerobic digester, one OTU (ID 837605) classified to the order of Bacteroidales became dominant, reaching a maximum abundance when the monensin concentration in the substrate was 3 mg · liter^−1^ (Fig. 4D). Subsequently, the Bacteroidales OTU population began to decline. This decline was followed by an increase in the population of an unknown OTU (ID 820298) belonging to the order d153 in the phylum of OP11 (Microgenomates) (Fig. 4D). This OTU rapidly rose in abundance from <0.01% on day 301 (premonensin) to a maximum of 38.3% on day 369 (P4, high monensin) in the slow anaerobic digester (Fig. 4D), when the monensin concentration in the substrate was 5 mg · liter^−1^ (Table 1). This OP11 OTU was one of only two OP11 OTUs found in the anaerobic digester and represented the majority of the phylum of OP11. During the shift in OTUs, the performance (methane yield) of the slow anaerobic digester partially recovered (Fig. 2A). Therefore, the rising OTU (ID 820298) appeared to be redundant with OTUs belonging to the order of Bacteroidales (Fig. 4D; Fig. S3D). This shift from the Bacteroidales OTU to the OP11 OTU populations drove the divergence of the slow anaerobic digester microbiome along the PCo1 axis of the principal-coordinate analysis plot (Fig. 1A). In the final two time points sequenced from the slow anaerobic digester (days 381 and 383 at the end of period 4), this OP11 OTU population began to decline and the OTUs belonging to the Bacteroidales order, including OTU 837605, began to increase in abundance (Fig. 4D; Fig. S3D). The OP11 OTU also increased in the fast anaerobic digester after feeding with the direct-dosed manure but to a much lower extent than in the slow anaerobic digester—from not detectable on day 257 (P2, prior to high concentrations of monensin) to a maximum of 0.3% on day 306 (P3, high monensin) (Fig. 4C).

Other researchers have already reported the phylum of OP11 in anaerobic digestion, but generally as a rare species (30, 31). Recent work has shown that members of the OP11 phylum have genes that encode the complete degradation of cellulose (18) and are likely able to produce formate, acetate, ethanol, and lactate (32). A single-cell sequencing study within the phylum of OP11 showed the presence of antibiotic resistance, stress tolerance, and the possession of a Gram-negative cell wall (33). This OTU from OP11 may have antibiotic resistance and stress tolerance capabilities similar to those of the strain sequenced in the previous study, which enabled it to enter the metabolic niche previously filled by Bacteroidales OTUs. The OP11 OTU appears to have been an *r*-strategist, which is a fast-growing organism that is able to become dominant in an unstable environment (34), and appears to have replaced the Bacteroidales OTUs during the monensin disturbance in the slow anaerobic digester. However, it will take culturing of members within this OTU to conclusively state that it was redundant with OTUs belonging to the order Bacteroidales. The monensin concentrations were never decreased in our anaerobic digesters. Therefore, it is unknown whether the Bacteroidales OTUs would have recovered completely when the disturbance was lifted (resilience).

To rule out a new OTU (that was introduced with, for example, the manure) rather than a redundant OTU from the digester microbiome to explain the recovery of the methane yield for the slow anaerobic digester in P4 at the high monensin concentrations, we carried out a bipartite network analysis of OTU associations with samples (see Fig. S6A to D). The bipartite network analysis visualizes samples with similar OTU contents being placed close to each other, resulting in a chronological order of the samples through the entire operating period (Fig. S6A). The same bipartite network also identified the OTUs that were present during monensin exposure in P4 (late, yellow dots in Fig. S6B) and prior to monensin exposure in P1 to P3 (early, yellow dots in Fig. S6C). We also identified differences in populations (primarily in the Clostridia class or Bacteroidales order) that were exclusively present in the early and late microbiomes of the slow anaerobic digester and that did not include the OP11 and Bacteroidales OTUs (Fig. S6D). However, all of these OTUs were rare OTUs in the digester. The combined relative abundances of the 294 OTUs and 105 OTUs that were found to be exclusively present during P1 to P3 (early, Fig. S6C) and P4 (late, Fig. S6B) were lower than 0.7% and 1.3% in any one sample, respectively (data not shown). Thus, the shifts in composition in the slow anaerobic digester microbiome under high monensin concentrations primarily consisted of changes in the relative abundance of already present OTU populations (e.g., OP11), which is in line with our redundancy observation, rather than the emergence of new OTU populations.

### Anaerobic digester microbiomes all shifted upon the introduction of either direct-dosed monensin manure or manure from monensin feed-dosed cows.

We did not find significant changes in the richness and unevenness measurements for the anaerobic digester samples with monensin addition, for both the low A and low B digesters that received manure from the monensin feed-dosed cows and the fast and slow digesters that received direct-dosed monensin (at a significance level of *P* < 0.05) (Fig. S4 and S5). However, we did observe changes in the compositions of the anaerobic digester microbiomes with either the addition of the manure from the monensin feed-dosed cows (for the low A and low B anaerobic digesters) or the direct addition of monensin to the control cow manure substrate (for the fast and slow anaerobic digesters). This is visualized in the PCoA plot of the anaerobic digester microbiome samples (based on weighted UniFrac distance) (Fig. 1A). The slow anaerobic digester diverged the most compared to all microbiome samples, which is indicated by a clear divergence of the slow digester microbiome away from all the other digester samples along the PCo1 axis of the PCoA (Fig. 1A). However, all anaerobic digester microbiomes changed to some extent with monensin addition. This was also visualized in boxplots representing the variation of the anaerobic digester communities with monensin addition (Fig. 3A) and a plot representing the similarity of the disturbed anaerobic digester microbiomes (i.e., with monensin addition) to their predisturbance states (Fig. 3B). According to the results of a distance-based redundancy analysis (db-RDA; or capscale analysis in R), four different environmental and functional parameters best explained the variation seen in the anaerobic digester communities: (i) total ammonium concentrations in the anaerobic digester (environmental), (ii) the monensin concentrations in the substrates (environmental), (iii) bicarbonate alkalinity (environmental), and (iv) the measured methane yields (function) (Fig. 1B).

Some common patterns were observed in terms of the shifts in the taxonomic compositions of all the anaerobic digesters when monensin was introduced. Dominant OTUs within the order Bacteroidales (phylum Bacteroidetes) underwent a notable succession event corresponding with the start of monensin addition in all anaerobic digesters (Fig. 4A to D; Table S7 and S8). Bacteroidetes members are Gram-negative acidogenic bacteria, which have been shown to play important roles in polysaccharide and protein degradation and are commonly found in anaerobic digesters (31, 35–37). They are also generalists, which seem to be easily replaceable with other generalists (38). Werner et al. (39) observed in an anaerobic digester microbiome study that acidogenic members in the phylum Bacteroidetes relied on redundancy during a disturbance to maintain overall community function. Upon returning to predisturbance conditions, Werner et al. (39) noted that the original Bacteroidetes populations did not return. Thus, OTUs in the phylum of Bacteroidetes were not found to be compositionally resilient in the anaerobic digesters.

We also found that specialists within syntrophic acetogenic bacterial populations either reduced or increased their abundance during the introduction of monensin in all anaerobic digesters. An OTU (ID 675613) belonging to the genus Pelotomaculum, which is a syntrophic propionate oxidizer within the order Clostridiales (40), was found to be positively correlated with monensin addition in all the anaerobic digesters (Table S7). On the other hand, three syntrophic OTUs (IDs 562603, 736989, and 111131), of which two belonged to the syntrophic fatty acid-degrading genus of Syntrophus (41), as well as one belonging to the syntrophic amino acid-degrading genus “Candidatus Cloacamonas” (phylum of WWE1) (42), were found to be negatively correlated with monensin concentrations in all anaerobic digesters (*P* < 0.001) (Table S8). The genus “Candidatus Cloacamonas” was previously found to be sensitive to changes in anaerobic digester conditions (43). Monensin has decreased the precursors of methanogenesis in the cow rumen by targeting hydrogen-, formate-, and acetate-producing bacteria and increasing propionate-producing bacteria (5), and thus the effect of the syntrophic bacterial populations may be pertinent. Besides the acidogenic and syntrophic acetogenic populations, OTUs belonging to the genus *HA73* within the family Desulfovibrionaceae were positively correlated in all digesters (see Tables S7 and S8).

## DISCUSSION

Whether through direct or indirect mechanisms, monensin impacted the microbiomes and performance of all the anaerobic digesters of this study. Some common shifts were observed in the acidogenic and syntrophic acetogenic populations in all digesters with monensin addition. The manure from monensin feed-dosed cows that was fed to the digesters had low concentrations of monensin (less than 0.2 mg · liter^−1^). Despite this, a shift was observed in the microbiomes and the performance of the anaerobic digesters that received manure from monensin feed-dosed cows. However, we attributed this shift to chemical and/or microbial differences in this manure versus the manure from the control cows rather than to the monensin itself. However, the rapid direct introduction of higher concentrations of monensin (up to 5 mg · liter^−1^) led to the functional instability of the anaerobic digester microbiome. On the other hand, a slower direct introduction of the same concentrations of monensin enabled another anaerobic digester microbiome to adapt and recover its performance. In both of the direct-dosed anaerobic digesters, rare OTUs rapidly rose in abundance during inhibitory high monensin concentrations. In the anaerobic digester that was able to adapt to monensin, an OP11 OTU appeared to be redundant with members of the Bacteroidales order because of the observed functional stability during this severe disturbance.

Thus, we observed that redundancy in the anaerobic digestion microbiome is a pertinent microbial mechanism behind the functional stability during a severe disturbance with the antibiotic monensin, albeit that enough operating time was required to adapt. This study builds upon previous studies in demonstrating the importance of redundancy in maintaining stability in anaerobic digesters. When digesters are operated properly, several studies have shown that anaerobic digester microbiomes are functionally stable by providing a diverse pool of redundant members to recover from disturbances (3, 44, 45). We performed a time series study of anaerobic digestion microbiomes to both characterize the functional stability of the microbiome and to explore how that microbiome shifts in response to a disturbance. Microbial ecologists also want to understand the temporal variability inherent in microbial communities, because then they may be able to predict how communities will respond to disturbances (46). However, studies looking at stability as related to natural environments often are faced with numerous variables that are difficult to control. In contrast, controlled engineered environments, such as anaerobic digesters, represent ideal systems to carry out time series studies examining the effects of disturbances on the microbiome (21, 39, 47). Therefore, we believe that our outcomes should be translatable to microbial ecology research on natural environments. However, some caveats of this study still exist: (i) duplicate sequencing of individual samples was not performed; (ii) duplicate operations for the fast and slow bioreactors were not performed, and therefore we relied on the time series analysis of each microbiome and the observed predictability of the anaerobic digester microbiome in this study; and (iii) Illumina 16S rRNA gene sequencing with one primer set was the only technique used. Future work should look at how monensin and other antibiotics impact the activity (function) of the anaerobic digester microbiome.

## MATERIALS AND METHODS

### Monensin dosing to cows.

Seven dairy cows (Cornell University Teaching and Research Center, Dryden, NY) were split into two groups and fed the same diet, except that three cows (monensin feed-dosed cows) received a ration top-dressed with monensin (Rumensin 90 Premix [Elanco Animal Health, Indianapolis, IN] mixed with corn grain), while four cows (control cows) received no monensin (ration top-dressed with corn grain without Rumensin). The monensin dosing rate in the top-dressing was increased every 2 weeks for a period of 2 months. The monensin concentrations in the top-dressing were later measured by liquid chromatography with postcolumn derivatization, as described by Coleman et al. (48) (Covance Laboratories, Greenfield, IN). On the basis of the measurements of the top-dressing concentrations, the monensin dosing rates were calculated to be approximately 194, 320, 432, and 546 mg · day^−1^ on a per-cow basis. The use of dairy cows in this study was in accordance with the recommendations of the Cornell Institutional Animal Care and Use Committee under the Animal Welfare Assurance A3347-01 on file with the Office of Laboratory Animal Welfare (OLAW). The protocol used in this study was approved by the Cornell Institutional Animal Care and Use Committee.

### Anaerobic digester operation.

The manure from the two groups of dairy cows was collected at the end of each monensin dose period to obtain five batches of manure. The manure batches were aliquoted into 1-liter plastic containers and were stored at −20°C until several days prior to being fed to the anaerobic digesters (at which point the manure was transferred to a 4°C refrigerator to thaw). Four 4.5-liter continuously stirred anaerobic digesters were inoculated with an inoculum microbiome that consisted of a mixture of (i) ∼3.5 liters of digester sludge collected from anaerobic digester-treated municipal wastewater located at the Ithaca Area Wastewater Treatment Facility (Ithaca, NY), (ii) ∼0.1 liters of centrifuged solids from a lab-scale continuously stirred anaerobic digester, and (iii) and ∼0.9 liters of blended upflow anaerobic sludge blanket (UASB) anaerobic digester granules from a brewery treatment plant (Budweiser Anheuser-Busch InBev, Baldwinsville, NY). The four anaerobic digesters were then semicontinuously fed (every 2 days) with manure at a target organic loading rate of 2 g VS · liter^−1^ · day^−1^ and a 25-day hydraulic retention time. We operated the anaerobic digesters at a temperature of 37 ± 1°C. To account for the possible effect of adding methanol in the substrate, 0.1 ml of methanol (with or without monensin) was added to all of the substrates in the study.

### Analytical methods.

Anaerobic digester performance and stability parameters (i.e., pH, tVFA concentrations, iVFA concentrations, total alkalinity, VS and total solids [TS], soluble chemical oxygen demand [COD] concentrations, total ammonium concentrations, biogas production rate, and biogas composition) were monitored routinely. In addition, the manure substrate was characterized (i.e., pH, gross energy, tVFA and iVFA concentrations, VS and TS concentrations, soluble and total COD concentrations, total alkalinity, and total ammonium concentrations). All analyses were carried out according to the *Standard Methods for the Examination of Water and Wastewater*, 21st ed. (49), unless noted otherwise. Biogas composition was measured using a gas chromatograph (SRI 8610C; SRI Instruments, Torrance, CA), which was equipped with a thermal conductivity detector (TCD) under isothermal conditions (i.e., 105°C) and a packed column (0.3-m HaySep-D packed Teflon; Restek, Bellefonte, PA) with helium as a carrier gas. iVFA concentrations were measured using a gas chromatograph (HP Hewlett Packard 5890 Series II), which was equipped with a flame ionization detector (FID) with a ramp temperature program (initial temperature, 70°C for 2 min; temperature ramp, 12°C per min to 200°C; final temperature, 200°C for 2 min) and a capillary column (NUKOL, fused silica capillary column, 15 m by 0.53 mm, 0.50-μm film thickness; Supelco Inc., Bellefonte, PA). The injection port was set to 200°C and the detector to 275°C. The gross energy of the manure was quantified via bomb calorimetry analysis by an independent laboratory (Dairy One, Ithaca, NY) using an IKA C2000 basic calorimeter system (IKA Works, Inc., Wilmington, NC). Effluent samples (200-ml volume) were collected from the anaerobic digesters monthly beginning during P1 and stored in a −23°C freezer for later analysis of monensin concentrations. As with the monensin concentrations in the top-dressing described above, liquid chromatography with postcolumn derivatization was used to measure monensin concentrations in the manure substrate and anaerobic digester effluent samples. Loss-on-drying analysis (105°C) was used to measure the moisture contents of the manure and effluent samples. The monensin concentrations in the wet manure and digester samples, as well as the moisture contents of the samples, were then used to calculate the concentrations of monensin in the samples on a dry matter basis. Monensin and loss-on-drying analyses were performed by Covance Laboratories (Greenfield, IN).

### Biomass sampling.

Biomass samples for 16S rRNA gene sequencing analysis were collected from (i) the hindguts of the dairy cows via rectal grab sampling, (ii) the manure substrates used for feeding the anaerobic digesters, and (iii) the anaerobic digesters. The hindgut biomass samples (184 samples) were collected from control cows and monensin feed-dosed cows approximately weekly during a 3-month period (i.e., for 1 month prior to the start of monensin dosing of the cows and for the 2 months during which a subset of the cows were subjected to monensin dosing). Duplicate samples were collected from each of the combined manures that were used as the substrates for the anaerobic digesters (10 samples). Finally, biomass samples were collected from the anaerobic digesters on an approximately weekly basis (154 samples). Samples were centrifuged, and 2 to 3 g of the solids was stored at −80°C for 16S rRNA gene sequencing analysis. In addition, we report details of the specific operating periods in which the samples were collected (see Table S9 in the supplemental material).

### DNA extraction, amplification, and sequencing.

DNA was extracted from the cow hindgut, manure, and anaerobic digester biomass samples using the PowerSoil DNA isolation kit (MoBio, Carlsbad, CA, USA). DNA extracted from the cow hindgut biomass samples was sent to the Earth Microbiome Project at the University of Colorado Boulder for further sample processing (i.e., PCR amplification via universal primers targeting the V4 region of the 16S rRNA gene—515F forward primer and 806R reverse primer—amplicon cleanup, and Illumina HiSeq 2000 sequencing). Details of the sample processing can be found at www.earthmicrobiome.org (50).

For the cow manure and anaerobic digester samples, we employed a modified version of the Earth Microbiome Project protocol (50). The modified protocol was outlined previously by Regueiro et al. (51), with the exception that in this study, 30 PCR cycles were used instead of 25. As in the study by Regueiro et al. (51), we performed duplicate PCRs of the extracted DNA samples and pooled the resulting amplicons prior to sequencing. Samples were sent for paired-end sequencing (2 × 250 bp) on the Illumina MiSeq platform (Illumina, San Diego, CA, USA) at the Cornell University Biotechnology Resource Center (Ithaca, NY, USA).

### Sequencing data analysis.

Paired-end reads were joined and then further processed via the Quantitative Insights into Microbial Ecology platform (QIIME 1.7) (52). Quality filtering was performed using the default values in QIIME with the exception that the minimum acceptable Phred quality score was set to 25. After demultiplexing, closed reference OTU picking with the default uclust method (53) was used to group sequences into operational taxonomic units (OTUs) at 97% identity. Taxonomy was assigned with the RDP classifier (54) using the Greengenes database (May 2013) for representative sequences selected for each OTU (55). This resulted in 8,330 OTUs. Approximately 20% of the initial 11.8 million quality-filtered sequences were discarded because they did not match any sequences in the reference database. Specifically, 16.1% ± 5.9% of the sequences from the manure samples, 20.2% ± 4.1% of the sequences from the low A digester samples, 19.8% ± 3.6% of the sequences from the low B digester samples, 20.4% ± 2.3% of sequences from the fast digester samples, and 19.6% ± 4.5% of sequences from the slow digester samples did not match sequences in the reference database. The average numbers of sequences per sample that failed to matched the reference database were not significantly different (*P* = 0.781) for the four anaerobic digesters, but the average numbers of failed sequences were significantly different (*P* = 0.030) between the manure and digester samples. Singletons were removed from the data set, resulting in 6,694 OTUs (minimum sequences per sample, 10,333; mean sequences per sample, 52,000). Alpha diversity was analyzed via the observed species (i.e., richness) and Gini coefficient metrics (i.e., unevenness, where 0 is equivalent to perfectly even and 1 is equivalent to uneven, with one abundant OTU and all other OTUs as singletons). Ten rarefactions at a depth of 10,300 sequences per sample were performed and collated. The weighted and unweighted UniFrac distance metrics (56) were used to analyze β diversity at an even sampling depth of 10,300 sequences per sample. Principal-coordinate analyses (PCoAs) were used to visualize the differences in communities between the samples. A bipartite network analysis examining the associations between OTUs and digester samples was carried out using the make_bipartite_network.py script in QIIME. Cytoscape v.3.5.1 was used to visualize the bipartite network analysis, using the spring-embedded layout model available in Cytoscape (57).

### Statistical analysis.

Constrained ordination, specifically, a distance-based redundancy analysis (db-RDA), was carried out using the capscale function in the package vegan in R (58). For the distance-based redundancy analysis, an analysis of variance (ANOVA) and the variance inflation factor (VIF) were used to select the environmental and functional parameters that best recreated the sample clustering observed in the PCoA plots. The variables included in the analysis were bicarbonate alkalinity, soluble COD concentrations, total ammonium concentrations, monensin concentrations in the substrate, specific biogas production rate, methane yields, pH of the effluents and substrates, VS and TS concentrations, and tVFA concentrations. Spearman's rank correlation coefficient was used in R (Hmisc package) to examine the correlation between monensin substrate concentrations and the relative abundance of the OTU populations. Only OTUs that reached at least 1% relative abundance in any one anaerobic digester sample were considered in the correlation analysis. Correlations were considered significant for *P* values of <0.001 (ρ < −0.5 for negative correlations and ρ > 0.5 for positive correlations). Statistical analyses of the environmental data were performed using the Tukey HSD model for comparing multiple means by pairwise comparisons in RStudio (v0.96.316).

### Accession number(s).

All amplicon and metadata have been made public through the QIITA data portal (qiita.microbio.me) under study number 1621 for the cow hindgut samples and study number 10560 for the cow manure and anaerobic digester biomass samples. Sequences were also submitted to EBI under the accession number ERP017357.

## Supplementary Material

Supplemental material
