# Supplementary material for: Redundancy in Anaerobic Digestion Microbiomes during Disturbances by the Antibiotic Monensin
Source: Appl Environ Microbiol. 2018 Apr 16;84(9):e02692-17. doi: 10.1128/AEM.02692-17 (PMC5930344; doi:10.1128/AEM.02692-17)
Supplement: Supplemental material [file supp_84_9_e02692-17__index.html]

Supplemental material 

# Redundancy in Anaerobic Digestion Microbiomes during Disturbances by the Antibiotic Monensin

## Supplemental material

- Supplemental file 1 -

  Monensin concentrations (Fig. S1); β-diversities of gut microbiome samples from cow hindguts from the control cows and monensin-dosed cows (Fig. S2); phyla that reached over 10% relative abundance in any one anaerobic digester sample from day 175 to the end of the operating period (Fig. S3); average observed richness metric (Fig. S4); averaged unevenness metric (Fig. S5); bipartite network analysis of OTU associations with samples (Fig. S6); physical and chemical data from manure characterization (Table S1); relative abundance of taxa (Tables S2 and S3); performance parameters measured for each anaerobic digester (Table S4); stability parameters (Table S5); individual VFA concentrations (Table S6); OTUs positively (Table S7) and negatively (Table S8) correlated with monensin concentrations in the substrate; anaerobic digester biomass samples (Table S9).

  PDF, 1.4M
