# Supplementary material for: Redundancy in Anaerobic Digestion Microbiomes during Disturbances by the Antibiotic Monensin
Source: Appl Environ Microbiol. 2018 Apr 16;84(9):e02692-17. doi: 10.1128/AEM.02692-17 (PMC5930344; doi:10.1128/AEM.02692-17)
Supplement: Supplemental material [file AEM.02692-17_zam009188496s1.pdf]

Supplementary Material

Redundancy of anaerobic digestion microbiomes during disturbances by the antibiotic monensin

Running Title: Monensin impact on stability of digester microbiomes

Catherine M. Spirito[1], Sarah E. Daly[1], Jeffrey J. Werner[2], Largus T. Angenent[1,3*]

[1] Department of Biological and Environmental Engineering, Cornell University, Ithaca, NY, USA.

[2] Chemistry Department, SUNY-Cortland, Cortland, NY, USA

[3] Center for Applied Geosciences, University of Tübingen, Tübingen, Germany

*Correspondence to:

L. T. Angenent

Center for Applied GeoSciences | University of Tübingen | Hölderlinstr. 12, 72074 Tübingen, Germany | Ph. +49-7071-601-322 | l.angenent@uni-tuebingen.de

Figures

Figure Captions

Figure S1: Monensin concentration measured in manure *vs.* monensin concentration measured in the consumed cow feed (on a dry matter basis in mg·kg^{-1}). The monensin excretion rate for the cows was calculated as the slope of the best-fit line for the plot.

Figure S2: β-diversity of gut microbiome samples from cow hindguts from the control cows and monensin-dosed cows: **A)** PCoA based on unweighted Unifrac distance. The samples were collected starting in the two weeks prior to monensin dosing through to end of the monensin dosing period (three months total). The samples are colored based on which cow they were collected from. Square shapes represent samples collected when the cows were dosed with monensin, while circles represent time periods when the cows were not fed monensin; and **B)** PCoA of unweighted Unifrac distance of combined manure substrates that were fed to anaerobic digesters. Red shading represents the concentration of monensin measured in the manure (M0, M200, M300, M400, and M500).

Figure S3: Plot of phyla that reached over 10% relative abundance in any one anaerobic digester sample from Day 175 to the end of the operating period for each of the anaerobic digesters: **A)** Low A; **B)** Low B; **C)** Fast; and **D)** Slow anaerobic digester. Shading represents periods.

Figure S4: Averaged observed OTUs (richness) metric during different periods for each of the anaerobic digesters: **A)** Low A; **B)** Low B; **C)** Fast; and **D)** Slow anaerobic digester.

Figure S5: Averaged Gini coefficient (unevenness) metric during different periods for each of the anaerobic digesters: **A)** Low A; **B)** Low B; **C)** Fast; and **D)** Slow anaerobic digester.

Figure S6: Bipartite network analysis of OTU associations with samples throughout the operating period of the Slow reactor, with samples colored light to dark purple chronologically (**A**). We looked separately at OTUs associated with the "Late" final eight time points of P4 from Day 329 to 383 (**B**; highlighted in yellow) and the "Early" P1-P3 samples from Day 115 to 306 (**C**; highlighted in yellow). Intersecting OTUs that appeared in both the Early and Late samples were removed, and the taxonomies of OTUs exclusive to either early or late samples were summarized (**D**), according to major taxonomic groupings that exceeded 2% relative abundance. Abundance in the transitional time points between Day 307 to 328 (not highlighted) were left out of the averages. The network visualization was created using the spring-embedded layout model in Cytoscape, using only OTUs that appeared in more than two samples and fewer than 66% of the samples.

Figure S1

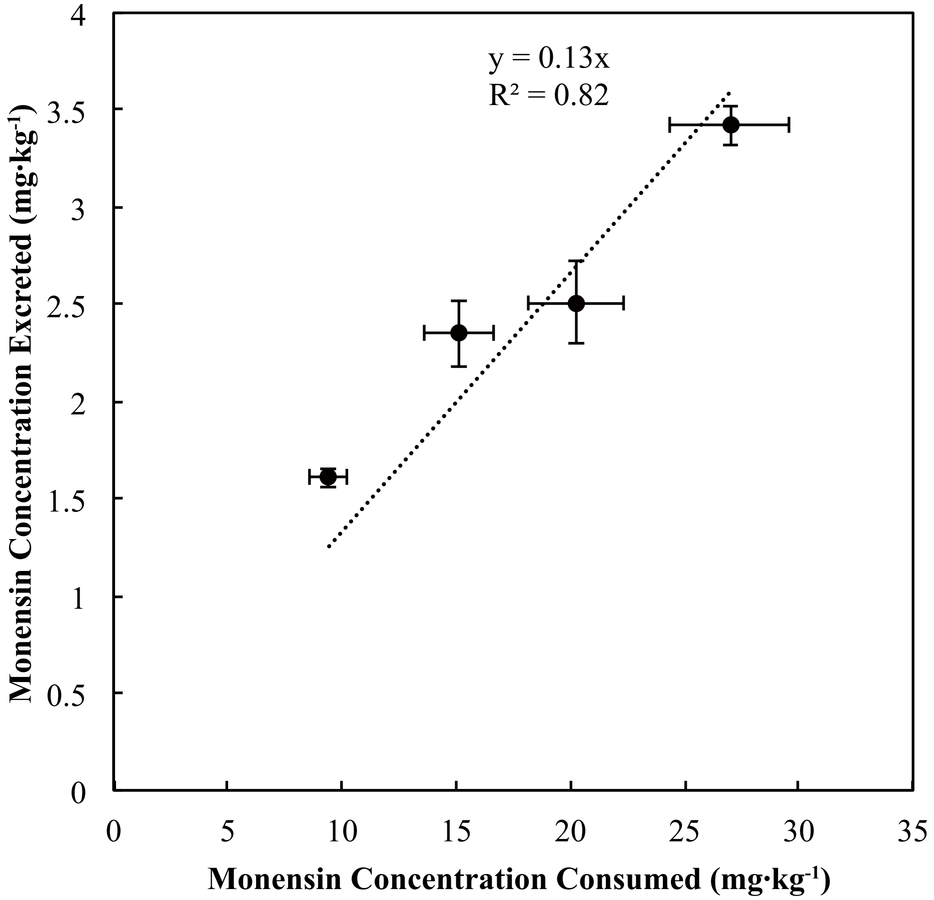

y = 0.13x
R² = 0.82
Monensin Concentration Excreted (mg·kg-1)
Monensin Concentration Consumed (mg·kg-1)
0
0.5
1
1.5
2
2.5
3
3.5
4
0
5
10
15
20
25
30
35

Figure S2

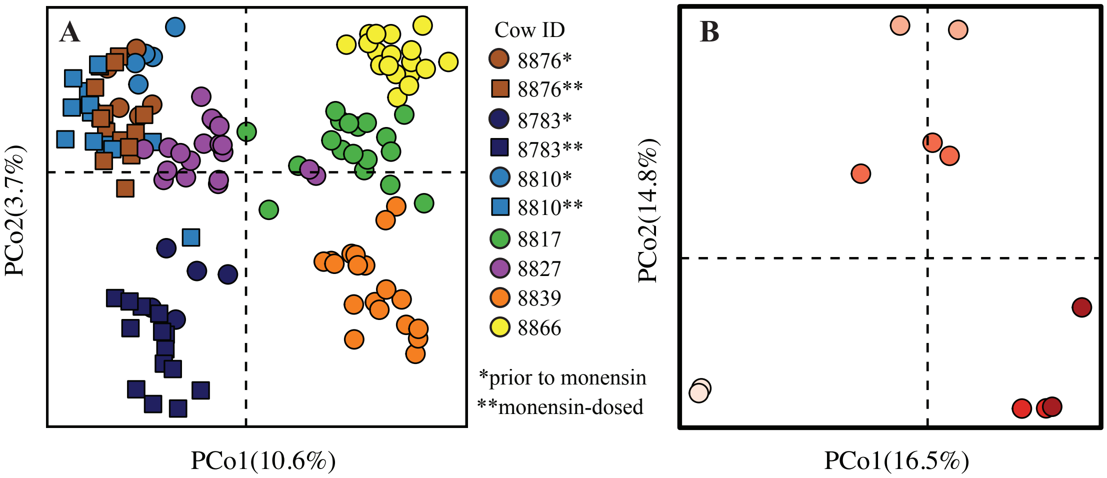
A
Cow ID
8876*
8876**
8783*
8783**
8810*
8810**
8817
8827
8839
8866
*prior to monensin
**monensin-dosed
PCo2(3.7%)
PCo1(10.6%)
B
PCo2(14.8%)
PCo1(16.5%)

Figure S3

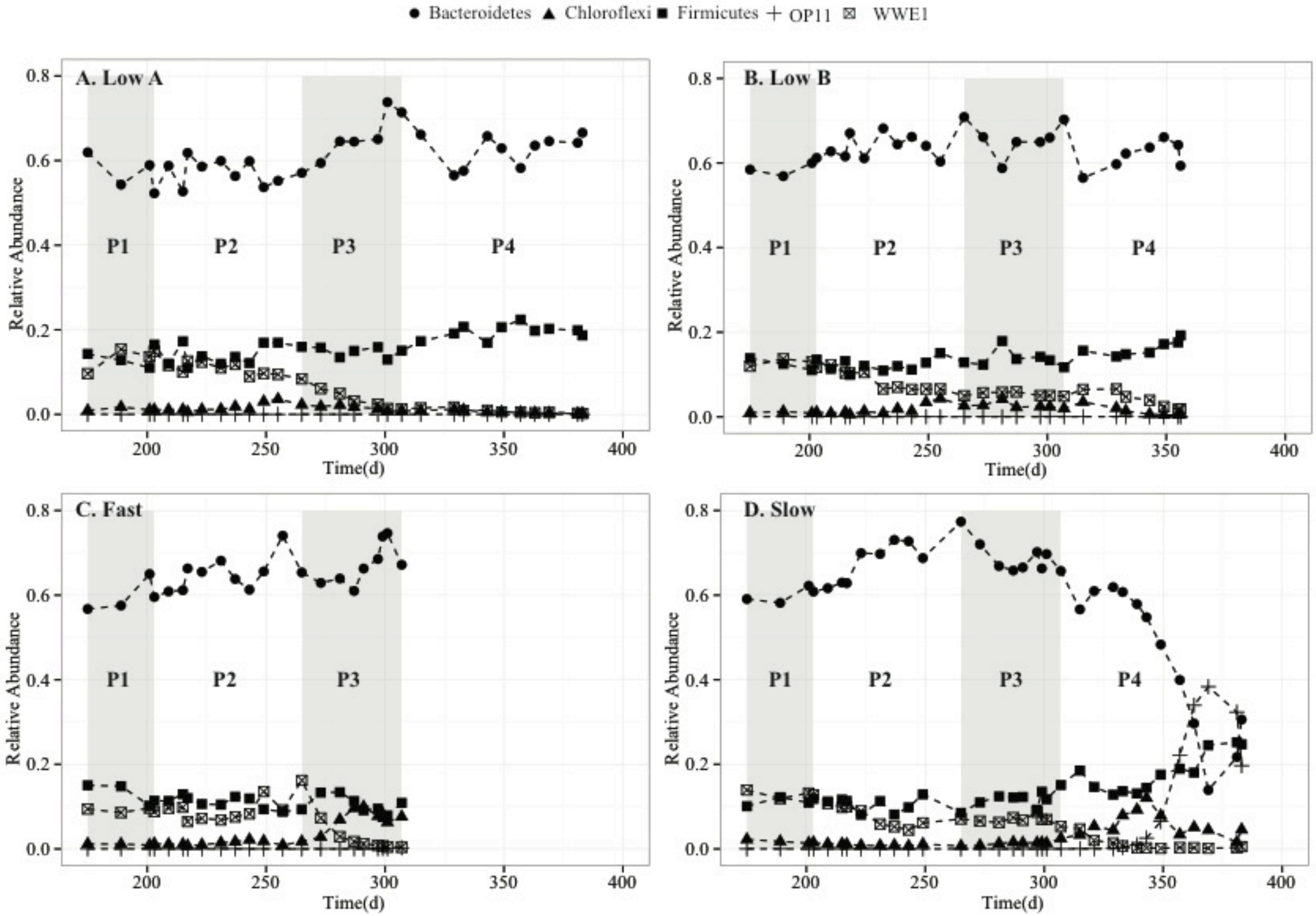

Bacteroidetes
Chloroflexi
Firmicutes
OP11
WWE1
A. Low A
B. Low B
C. Fast
D. Slow
P1
P2
P3
P4
Relative Abundance
Time(d)
0.0
0.2
0.4
0.6
0.8
200
250
300
350
400

Figure S4

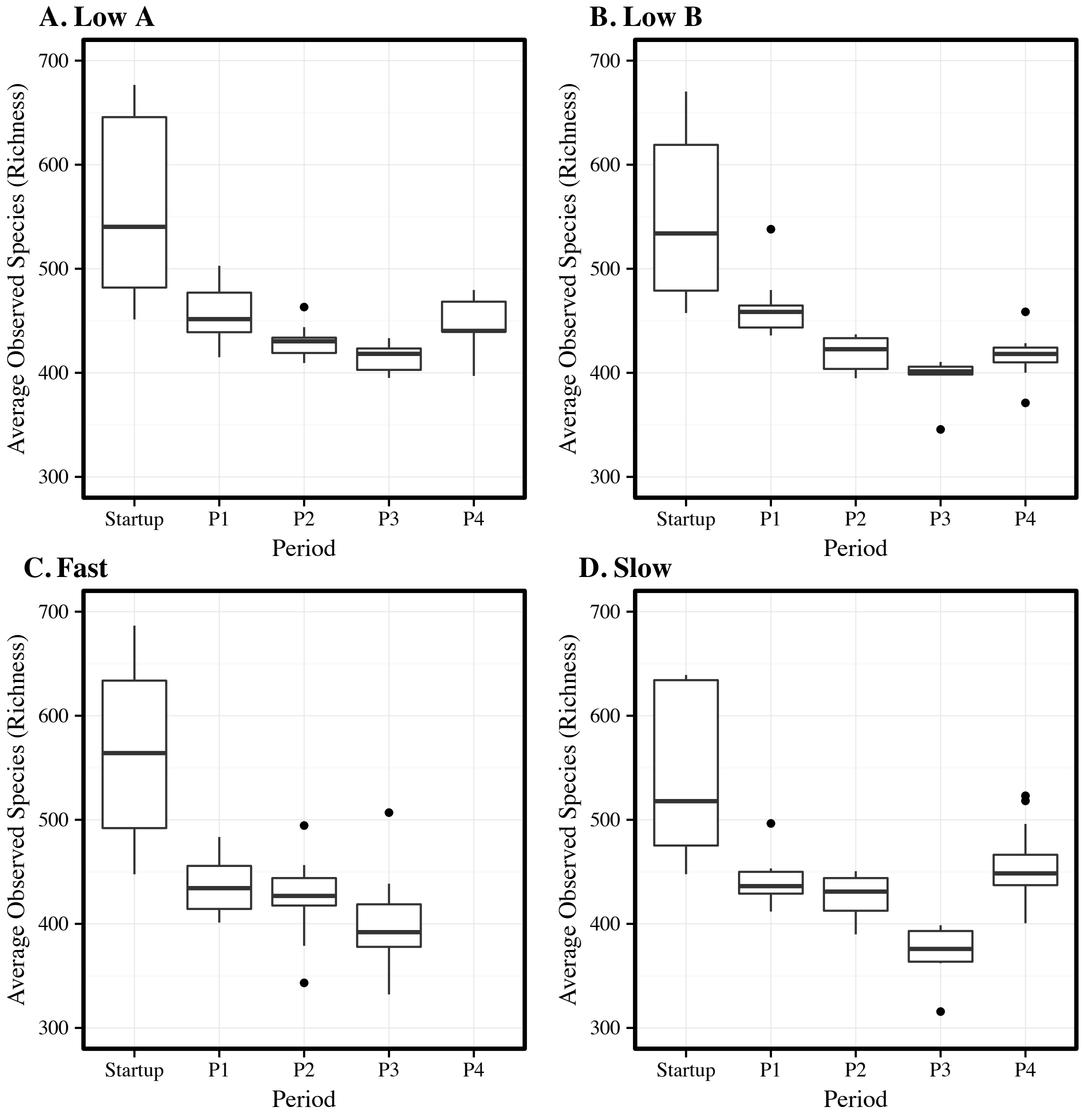
A. Low A
B. Low B
C. Fast
D. Slow
Average Observed Species (Richness)
300
400
500
600
700
Startup
P1
P2
P3
P4
Period

Figure S5

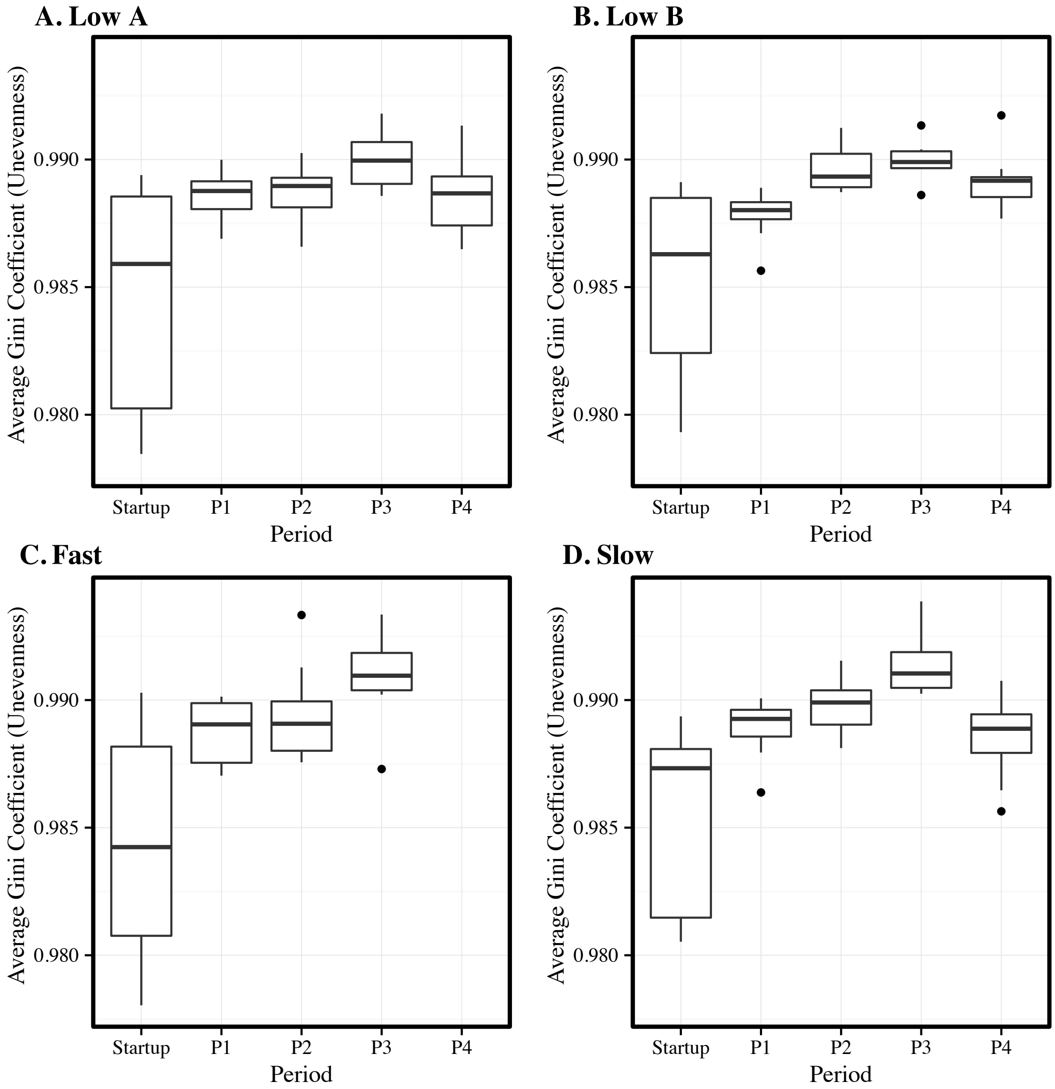
A. Low A
B. Low B
C. Fast
D. Slow
Average Gini Coefficient (Unevenness)
0.980
0.985
0.990
Startup
P1
P2
P3
P4
Period

Figure S6

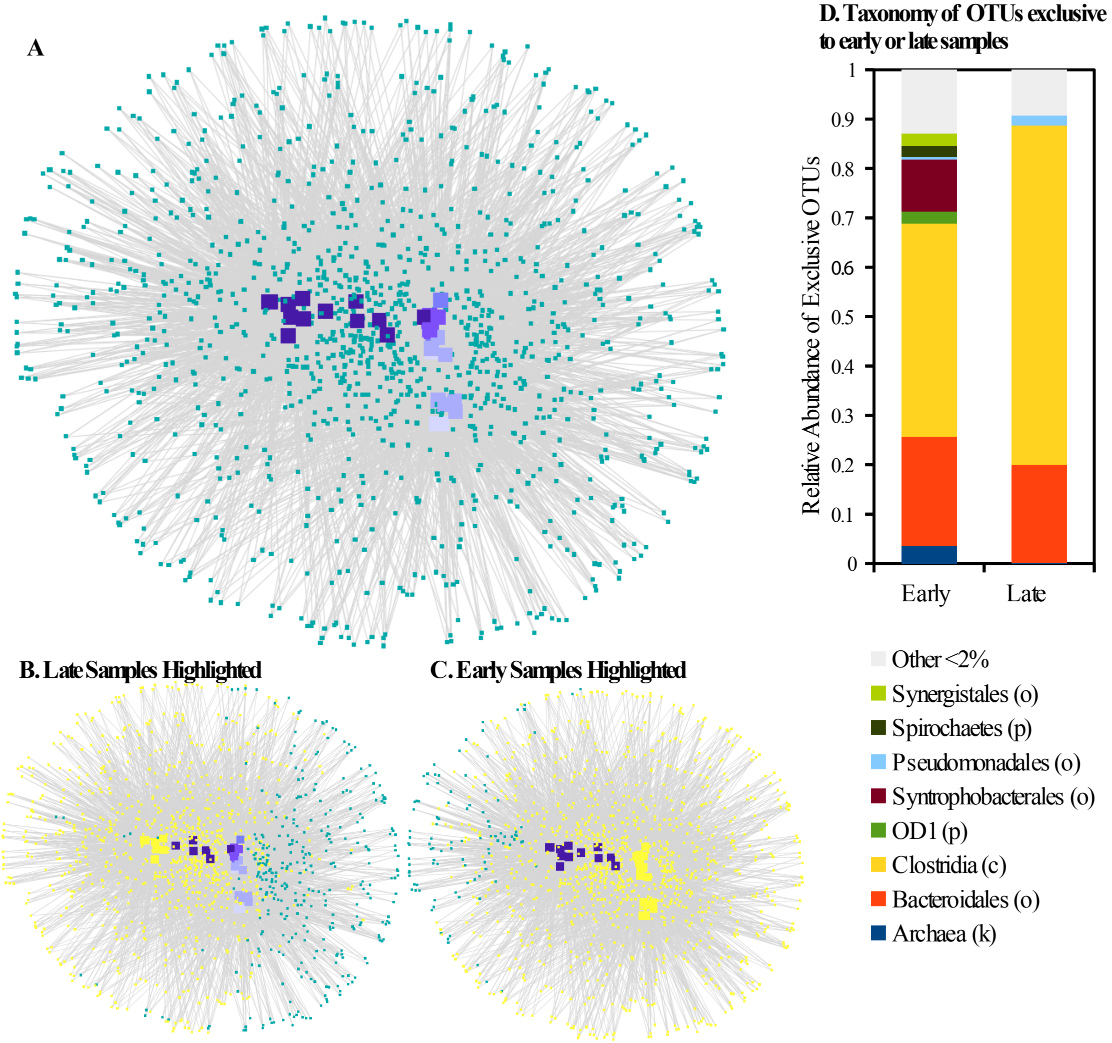
A
B. Late Samples Highlighted
C. Early Samples Highlighted
D. Taxonomy of OTUs exclusive to early or late samples
Relative Abundance of Exclusive OTUs
0
0.1
0.2
0.3
0.4
0.5
0.6
0.7
0.8
0.9
1
Early
Late
Other <2%
Synergistales (o)
Spirochaetes (p)
Pseudomonadales (o)
Syntrophobacterales (o)
OD1 (p)
Clostridia (c)
Bacteroidales (o)
Archaea (k)

Tables

Table Captions

Table S1: Physical and chemical data from manure characterization. Values are bold when significant differences from control manure values were observed ($p<0.05$). Star (*) indicates whether the monensin manure remained significantly different after considering the dilutions of the manures. Errors are represented as standard deviations.

Table S2: Relative abundance (%) of taxa families in the manure substrate samples. Only families that reached at least 1% relative abundance in any one manure substrate sample were included in the table. This resulted in 17 families that represented an average of 95.33% of the total composition of the manures. Taxa that were unclassified to the family level are included in the table (*i.e.,* Bacteroidales Unknown Family in table represents all OTUs that were classified to the order Bacteroidales but were unclassified at the family level). M0 refers to the manure from the control cows. M200, M300, M400, and M500 refer to the four batches of manure collected from the monensin-dosed cows (corresponding to the 194, 320, 432, and 546 $mg \cdot d^{-1}$ monensin-dosing rates, respectively). Duplicate or triplicate samples were run from each manure substrate and are shown in the table as separate entries.

Table S3: Average relative abundance (%) of taxa families in the seven digester inoculum (Day 0) samples. Only families that reached at least 1% relative abundance in any one digester inoculum sample are included in this table. Taxa that were unclassified to the family level are included in the table (*i.e.,* Bacteroidales Unknown Family in table represents all OTUs that were classified to the order Bacteroidales but were unclassified at the family level). This resulted in 20 families that represent 86.10% of the total composition of the inoculum samples.

Table S4: Performance parameters measured for each anaerobic digester. Errors are represented as standard deviations.

Table S5: Stability parameters measured for each anaerobic digester, reported as averages for each period. Errors are represented as standard deviations. The average total VFAs reported in this table were measured using the distillation method (5560C) outlined in the Standard Methods for the Examination of Water and Wastewater, 21st ed.

Table S6: Individual VFA (acetate, propionate, and *n*-butyrate) concentrations (mM) were measured by gas chromatography (gas chromatograph equipped with flame ionization detector; see main methods section). Total VFA concentrations ($mmol\ Ac \cdot L^{-1}$) were measured using the distillation method (5560C) outlined in the Standard Methods for the Examination of Water and Wastewater, 21st ed. Values in this table are individual measurements made on the day indicated.

Table S7: OTUs positively correlated (Spearman's rank correlation, $\rho > 0.5$, $p<0.001$) with monensin concentrations in the substrate for the Slow anaerobic digester from Day

175 on. Table also indicates if the OTU was positively correlated with monensin concentrations in the Fast anaerobic digester (Y=yes; N=no) and if it was positively correlated with monensin concentrations in all anaerobic digesters (*i.e.*, Low A, Low B, Fast, and Slow). Only OTUs that reached a min of 1% relative abundance in at least one anaerobic digester sample (from Day 175 on) are included.

Table S8: OTUs negatively correlated (Spearman's rank correlation, $\rho < -0.5$, $p < 0.001$) with monensin concentrations in substrate for the Slow anaerobic digester from Day 175 on. Table also indicates if the OTU was negatively correlated with monensin concentrations in the Fast anaerobic digester (Y=yes; N=no) and if it was negatively correlated with monensin concentrations in all anaerobic digesters (*i.e.*, Low A, Low B, Fast, and Slow). Only OTUs that reached a min of 1% relative abundance in at least one anaerobic digester sample (from Day 175 on) are included.

Table S9: Anaerobic digester biomass samples. In addition to these samples, ten manure substrate samples were sequenced (duplicate samples from each manure batch), as well as the cow hindgut samples.

Table S1

	Manures				
**Measured parameters**	**M0**	**M200**	**M300**	**M400**	**M500**
Dilution Factor (g raw manure per g diluted manure basis)	0.391	0.372	0.392	0.379	0.359
Monensin-Dosing Rate ($mg \cdot d^{-1}$)	ND	194 ± 1	320 ± 46	432 ± 19	546 ± 51
Monensin Concentration, wet matter basis ($mg \cdot L^{-1}$)	ND	0.239 ± 0.009	0.337 ± 0.008	0.371 ± 0.013	0.543 ± 0.011
Monensin Concentration, dry matter basis ($mg \cdot kg^{-1}$)	ND	1.610 ± 0.050	2.350 ± 0.170	2.510 ± 0.210	3.420 ± 0.100
pH	7.05 ± 0.22	7.19 ± 0.04	**7.58 ± 0.04***	**7.56 ± 0.11***	**7.55 ± 0.16***
Gross Energy ($cal \cdot g^{-1}$)	4590 ± 22	4607 ± 37	4583 ± 53	4543 ± 23	4612 ± 52
Total VFAs Concentration ($mg\ Ac \cdot L^{-1}$)	6416 ± 194	**5168 ± 125***	**5576 ± 338***	6212 ± 441	5861 ± 372*
Acetate/Propionate Ratio	7.3 ± 0.1	6.5 ± 0.5	7.0 ± 0.2	**9.0 ± 0.4***	8.0 ± 0.7
TS Concentration ($g \cdot L^{-1}$)	140 ± 1.9	**143.3 ± 4.7**	142.2 ± 1.6	**144.2 ± 2.5**	**156.1 ± 3**
VS Concentration ($g \cdot L^{-1}$)	126.8 ± 2.1	**130.0 ± 4.8**	127.0 ± 1.6	129.7 ± 2.5	**141.3 ± 3.0**
Total COD Concentration ($g\ O_2 \cdot L^{-1}$)	135.07 ± 19.47	146.00 ± 18.77	145.60 ± 14.35	118.40 ± 18.88	136.80 ± 13.49
Soluble COD Concentration ($g\ O_2 \cdot L^{-1}$)	24.40 ± 1.31	23.09 ± 0.72	26.67 ± 1.34	24.27 ± 2.36	23.20 ± 3.47
Total Alkalinity ($g\ CaCO_3 \cdot L^{-1}$)	9.3 ± 0.1	**10.4 ± 0.1***	**12.6 ± 0.3***	**12.5 ± 0.1***	**12.4 ± 0.1***
Total Ammonium Concentration ($g\ N \cdot L^{-1}$)	1.00 ± 0.04	0.90 ± 0.04	**1.44 ± 0.05***	**1.82 ± 0.18***	**1.97 ± 0.12***

Table S2

Family Name	Relative Abundance of Taxa Family in Manures (%)										
	**M0**	**M0**	**M200**	**M200**	**M300**	**M300**	**M300**	**M400**	**M400**	**M500**	**M500**
Aerococcaceae	0.81	1.55	1.50	1.59	9.09	8.95	1.29	16.95	17.09	19.24	21.26
Bacteroidaceae	1.32	1.18	1.03	0.99	1.57	1.21	1.80	0.92	0.96	0.52	0.77
Bacteroidales Family S24-7	4.48	3.68	2.76	2.65	3.09	2.44	3.22	1.55	1.67	1.61	1.70
Bacteroidales Unknown Family	8.35	10.81	4.76	3.97	8.09	5.73	7.60	1.99	2.45	2.57	3.33
Bifidobacteriaceae	5.67	5.12	0.10	0.10	0.06	0.07	0.06	1.47	1.11	0.84	0.88
Carnobacteriaceae	0.61	3.60	1.54	1.81	13.20	21.67	1.74	19.48	19.59	19.63	15.14
Clostridiaceae	4.85	4.17	7.82	8.53	3.57	2.82	5.77	3.03	2.90	5.52	6.25
Clostridiales Unknown Family	10.07	9.17	17.45	17.70	9.40	8.05	11.96	8.43	8.47	6.74	6.74
Erysipelotrichaceae	1.22	0.73	0.49	0.36	0.81	0.58	0.75	0.56	0.49	0.37	0.38
Lachnospiraceae	7.50	6.85	6.48	7.84	6.49	5.30	8.78	6.07	5.89	7.04	5.64
Methanobacteriaceae	1.73	1.76	0.77	0.62	1.04	0.74	0.71	0.81	0.59	1.12	1.10
Mollicutes RF39 Order, Unknown Family	5.95	6.20	10.15	9.12	5.49	5.24	5.49	4.84	4.88	3.08	3.52
Moraxellaceae	0.37	0.32	0.25	0.24	0.08	0.08	0.10	0.44	0.54	1.32	0.84
Rikenellaceae	0.99	0.83	0.96	0.89	1.36	1.15	1.40	0.49	0.59	0.37	0.55
Ruminococcaceae	41.17	39.55	38.74	38.48	30.01	30.21	43.55	27.47	27.76	24.25	25.57
Staphylococcaceae	0.03	0.07	0.28	0.29	0.24	0.25	0.12	0.67	0.54	0.69	1.25
Turicibacteraceae	0.45	0.44	0.08	0.09	1.26	0.90	0.89	0.17	0.11	0.14	0.16

Table S3

Taxa Family	Percent Average Relative Abundance (mean±sd)
Anaerolinaceae	6.27±0.69
Bacteroidales Family SB-1	0.96±0.09
Bacteroidales Unknown Family	26.00±1.58
Cloacamonaceae	9.23±1.26
Clostridia order SHA-98, Unknown Family	2.69±0.43
Clostridia Unknown Family	1.01±0.10
Comamonadaceae	1.79±0.26
Geobacteraceae	2.66±0.40
Methanobacteriaceae	7.86±1.33
Methanosaetaceae	1.41±0.29
Moraxellaceae	2.50±1.66
OP8 Candidate Phylum, Class OP8_2, Unknown Family	1.12±0.18
Pseudomonadaceae	3.28±1.42
Spirochaetaceae	5.60±1.53
Syntrophaceae	1.57±0.20
Syntrophobacteraceae	1.59±0.26
Syntrophomonadaceae	0.91±0.10
Tenericutes, class RF3, order ML615J-28, Unknown Family	1.70±0.27
Thermotogaceae	2.96±0.64
Tissierellaceae	4.98±0.96

Table S4

**Period**	**Startup (Days 1-114)**				**Period 1 (P1; Days 115-202)**			
Anaerobic digester	LowA	LowB	Fast	Slow	LowA	LowB	Fast	Slow
**Performance**								
Biogas Production Rate ($L \cdot L^{-1} \cdot d^{-1}$)	0.453 ± 0.061	0.467 ± 0.071	0.356 ± 0.124	0.444 ± 0.071	0.741 ± 0.072	0.76 ± 0.06	0.727 ± 0.056	0.718 ± 0.042
Specific Methane Yield ($L\ CH_4 \cdot gVS^{-1} d^{-1}$)	NA	NA	NA	NA	0.21 ± 0.014	0.211 ± 0.018	0.188 ± 0.017	0.195 ± 0.013
Methane Content (%)	NA	NA	NA	NA	54 ± 1	54 ± 1	54 ± 1	54 ± 1
VS Reduction Efficiency (%)	43.4 ± 4.4	42.7 ± 4.6	42 ± 4.2	42.7 ± 3.5	47.7 ± 4.1	47.6 ± 4.3	46.3 ± 2	49.5 ± 3.8
Soluble COD Reduction Efficiency (%)	NA	NA	NA	NA	63 ± 7	54 ± 22	65 ± 7	61 ± 11
**Period**	**Period 2 (P2; Days 203-264)**				**Period 3 (P3; Days 265-306)**			
Anaerobic digester	LowA	LowB	Fast	Slow	LowA	LowB	Fast	Slow
**Performance**								
Biogas Production Rate ($L \cdot L^{-1} \cdot d^{-1}$)	0.706 ± 0.11	0.716 ± 0.105	0.755 ± 0.043	0.732 ± 0.046	0.608 ± 0.032	0.653 ± 0.052	0.409 ± 0.275	0.722 ± 0.044
Specific Methane Yield ($L\ CH_4 \cdot gVS^{-1} d^{-1}$)	0.189 ± 0.027	0.192 ± 0.026	0.2 ± 0.013	0.195 ± 0.014	0.17 ± 0.009	0.18 ± 0.015	0.108 ± 0.086	0.2 ± 0.013
Methane Content (%)	54 ± 3	54 ± 3	53 ± 3	53 ± 2	56 ± 2	55 ± 1	44 ± 16	55 ± 2
VS Reduction Efficiency (%)	47.7 ± 4	46.4 ± 3.9	47 ± 4.5	47.4 ± 1.6	44.8 ± 5.1	48.1 ± 4	42.1 ± 6	48.1 ± 2.6
Soluble COD Reduction Efficiency (%)	64 ± 7	65 ± 5	67 ± 3	66 ± 5	57 ± 3	60 ± 4	37 ± 35	65 ± 5
**Period**	**Period 4 (P4; Days 307-383)**							
Anaerobic digester	LowA	LowB	Fast	Slow				
**Performance**								
Biogas Production Rate ($L \cdot L^{-1} \cdot d^{-1}$)	0.587 ± 0.033	0.636 ± 0.034		0.587 ± 0.075				
Specific Methane Yield ($L\ CH_4 \cdot gVS^{-1} d^{-1}$)	0.161 ± 0.009	0.176 ± 0.009		0.159 ± 0.022				

Methane Content (%)	55 ± 1	55 ± 1		55 ± 2	
VS Reduction Efficiency (%)	40.4 ± 3.7	42.1 ± 6		41.5 ± 3.6	
Soluble COD Reduction Efficiency (%)	50 ± 5	55 ± 5		57 ± 7	

Table S5

Period	Startup (Days 1-114)				Period 1 (P1; Days 115-202)			
Anaerobic digester	LowA	LowB	Fast	Slow	LowA	LowB	Fast	Slow
**Stability Parameters**								
pH	7.37 ± 0.07	7.38 ± 0.08	7.39 ± 0.08	7.38 ± 0.07	7.23 ± 0.09	7.23 ± 0.09	7.22 ± 0.08	7.23 ± 0.11
tVFA Concentration (mg Ac$\cdot$L^{-1})	184 ± 38	250 ± 123	237 ± 173	165 ± 39	161 ± 23	154 ± 21	130 ± 14	140 ± 28
Total Alkalinity (g $CaCO_3\cdot L^{-1}$)	NA	NA	NA	NA	5.7 ± 0.1	5.8 ± 0.1	5.8 ± 0.1	5.9 ± 0.1
Bicarbonate Alkalinity (g $CaCO_3\cdot L^{-1}$)	NA	NA	NA	NA	5.6 ± 0.1	5.7 ± 0.1	5.7 ± 0.1	5.8 ± 0
TS Concentration (g$\cdot$L^{-1})	38.3 ± 3.2	39.1 ± 3.9	39.7 ± 3.3	39.2 ± 2.8	31.5 ± 2.4	31.7 ± 2.3	32.2 ± 1.2	30.7 ± 2.2
VS Concentration (g$\cdot$L^{-1})	28.3 ± 2.2	28.7 ± 2.3	29 ± 2.1	28.7 ± 1.7	26.1 ± 2	26.2 ± 2.1	26.9 ± 1	25.3 ± 1.9
Soluble COD concentration (g $O_2\cdot L^{-1}$)	NA	NA	NA	NA	3.48 ± 0.66	4.43 ± 2.09	3.33 ± 0.63	3.72 ± 0.98
Total Ammonium concentration (g N$\cdot$L^{-1})	NA	NA	NA	NA	0.79 ± 0.01	0.81 ± 0.02	0.75 ± 0.05	0.84 ± 0.02
**Period**	**Period 2 (P2; Days 203-264)**				**Period 3 (P3; Days 265-306)**			
Anaerobic digester	LowA	LowB	Fast	Slow	LowA	LowB	Fast	Slow
**Stability Parameters**								
pH	7.22 ± 0.07	7.22 ± 0.08	7.17 ± 0.08	7.21 ± 0.09	7.22 ± 0.04	7.21 ± 0.05	6.71 ± 0.48	7.14 ± 0.07
tVFA Concentration (mg Ac$\cdot$L^{-1})	117 ± 23	119 ± 18	112 ± 23	107 ± 20	128 ± 15	122 ± 17	2473 ± 2426	108 ± 10
Total Alkalinity (g $CaCO_3\cdot L^{-1}$)	6.4 ± 0.5	6.3 ± 0.6	5.9 ± 0.1	5.9 ± 0.1	7 ± 0.1	6.9 ± 0.1	5.7 ± 0.4	6 ± 0.1
Bicarbonate Alkalinity (g $CaCO_3\cdot L^{-1}$)	6.3 ± 0.5	6.2 ± 0.6	5.8 ± 0.2	5.8 ± 0.1	6.9 ± 0.1	6.8 ± 0.1	3.8 ± 2.1	6 ± 0.1
TS Concentration (g$\cdot$L^{-1})	31 ± 2.5	32.1 ± 1.9	31.5 ± 2.3	31.1 ± 0.8	32.9 ± 2.7	31.3 ± 2.2	34.1 ± 3.1	30.9 ± 1.2
VS Concentration (g$\cdot$L^{-1})	26.1 ± 2	26.8 ± 2	26.5 ± 2.3	26.3 ± 0.8	27.7 ± 2.5	26 ± 2	29 ± 3	25.9 ± 1.4
Soluble COD concentration (g $O_2\cdot L^{-1}$)	3.29 ± 0.47	3.27 ± 0.3	3.09 ± 0.32	3.28 ± 0.45	3.63 ± 0.28	3.33 ± 0.34	7.34 ± 5.11	3.28 ± 0.5
Total Ammonium concentration (g N$\cdot$L^{-1})	0.95 ± 0.11	0.96 ± 0.11	0.85 ± 0.05	0.82 ± 0.04	1.11 ± 0.1	1.12 ± 0.08	0.96 ± 0.1	0.84 ± 0.05
**Period**	**Period 4 (P4; Days 307-383)**							
Anaerobic digester	LowA	LowB	Fast	Slow				

**Stability Parameters**					
pH	7.23 ± 0.08	7.22 ± 0.07		7.14 ± 0.1	
tVFA Concentration (mg $Ac \cdot L^{-1}$)	155 ± 29	162 ± 27		384 ± 446	
Total Alkalinity (g $CaCO_3 \cdot L^{-1}$)	7.2 ± 0.2	7.2 ± 0.1		6.3 ± 0.2	
Bicarbonate Alkalinity (g $CaCO_3 \cdot L^{-1}$)	7 ± 0.2	7.1 ± 0.1		6 ± 0.3	
TS Concentration ($g \cdot L^{-1}$)	35 ± 1.9	32.4 ± 1.2		34.1 ± 1.9	
VS Concentration ($g \cdot L^{-1}$)	29.8 ± 1.9	27.1 ± 1.1		29.2 ± 1.8	
Soluble COD concentration (g $O_2 \cdot L^{-1}$)	4.13 ± 0.38	3.77 ± 0.39		4.13 ± 0.69	
Total Ammonium concentration (g $N \cdot L^{-1}$)	1.13 ± 0.1	1.2 ± 0.14		0.9 ± 0.15	

Table S6

Anaerobic digester	Day	Acetate concentration (mM)	Propionate concentration (mM)	*n*-Butyrate concentration (mM)	tVFA concentration measured (mmol Ac·L^{-1})
Low A	219	0.24	ND	ND	1.69
Low A	245	0.20	ND	ND	1.69
Low A	285	0.14	ND	ND	1.69
Low A	345	0.11	ND	ND	3.17
Low B	247	0.13	ND	ND	1.79
Low B	323	0.17	ND	ND	2.34
Low B	333	0.11	ND	ND	2.34
Low B	345	0.42	ND	ND	3.01
Low B	353	0.90	ND	ND	3.39
Fast	243	0.62	0.45	ND	1.60
Fast	285	7.36	ND	ND	4.35
Fast	293	38.98	3.69	1.15	55.57
Fast	307	38.61	9.86	2.81	102.69
Slow	321	0.52	ND	ND	2.18
Slow	323	1.31	ND	ND	3.33
Slow	333	11.64	1.68	ND	28.61
Slow	345	0.56	ND	ND	3.33
Slow	353	0.38	ND	ND	2.72

Table S7

OTU ID	Taxonomy	Fast Digester	All Digesters
842598	k__Archaea; p__Euryarchaeota; c__Methanobacteria; o__Methanobacteriales; f__Methanobacteriaceae; g__Methanobrevibacter; s__	N	N
628811	k__Bacteria; p__Armatimonadetes; c__SJA-176; o__TP122; f__; g__; s__	N	N
837605	k__Bacteria; p__Bacteroidetes; c__Bacteroidia; o__Bacteroidales; f__; g__; s__	Y	Y
559410	k__Bacteria; p__Bacteroidetes; c__Bacteroidia; o__Bacteroidales; f__; g__; s__	N	N
4059526	k__Bacteria; p__Bacteroidetes; c__Bacteroidia; o__Bacteroidales; f__; g__; s__	N	N
4443296	k__Bacteria; p__Bacteroidetes; c__Bacteroidia; o__Bacteroidales; f__; g__; s__	N	N
1891861	k__Bacteria; p__Bacteroidetes; c__Bacteroidia; o__Bacteroidales; f__[Paraprevotellaceae]; g__YRC22; s__	N	N
541252	k__Bacteria; p__Bacteroidetes; c__Bacteroidia; o__Bacteroidales; f__Porphyromonadaceae; g__; s__	Y	Y
3506234	k__Bacteria; p__Bacteroidetes; c__Bacteroidia; o__Bacteroidales; f__Porphyromonadaceae; g__; s__	Y	Y
4383641	k__Bacteria; p__Bacteroidetes; c__Bacteroidia; o__Bacteroidales; f__Porphyromonadaceae; g__; s__	Y	Y
1764554	k__Bacteria; p__Bacteroidetes; c__Bacteroidia; o__Bacteroidales; f__Porphyromonadaceae; g__Parabacteroides; s__	N	N
258334	k__Bacteria; p__Chloroflexi; c__Anaerolineae; o__Anaerolineales; f__Anaerolinaceae; g__T78; s__	Y	N
809315	k__Bacteria; p__Chloroflexi; c__Anaerolineae; o__Anaerolineales; f__Anaerolinaceae; g__T78; s__	Y	N
591951	k__Bacteria; p__Firmicutes; c__Clostridia; o__Clostridiales; f__; g__; s__	N	N
178657	k__Bacteria; p__Firmicutes; c__Clostridia; o__Clostridiales; f__Clostridiaceae; g__Clostridium; s__	N	N
675613	k__Bacteria; p__Firmicutes; c__Clostridia; o__Clostridiales; f__Peptococcaceae; g__Pelotomaculum; s__	Y	Y
550485	k__Bacteria; p__Firmicutes; c__Clostridia; o__Clostridiales; f__Ruminococcaceae; g__; s__	N	N
582502	k__Bacteria; p__Firmicutes; c__Clostridia; o__Clostridiales; f__Ruminococcaceae; g__; s__	N	N
743075	k__Bacteria; p__Firmicutes; c__Clostridia; o__Clostridiales; f__Ruminococcaceae; g__; s__	N	N

539716	k__Bacteria; p__Firmicutes; c__Clostridia; o__Clostridiales; f__Syntrophomonadaceae; g__Syntrophomonas; s__	N	N
820298	k__Bacteria; p__OP11; c__WCHB1-64; o__d153; f__; g__; s__	N	N
286595	k__Bacteria; p__Proteobacteria; c__Deltaproteobacteria; o__Desulfovibrionales; f__Desulfomicrobiaceae; g__Desulfomicrobium; s__	N	N
4353504	k__Bacteria; p__Spirochaetes; c__Spirochaetes; o__Spirochaetales; f__Spirochaetaceae; g__Treponema; s__	N	N
767422	k__Bacteria; p__Synergistetes; c__Synergistia; o__Synergistales; f__Dethiosulfovibrionaceae; g__HA73; s__	Y	Y
703270	k__Bacteria; p__Synergistetes; c__Synergistia; o__Synergistales; f__Synergistaceae; g__vadinCA02; s__	N	N
616150	k__Bacteria; p__Verrucomicrobia; c__Opitutae; o__Puniceicoccales; f__Puniceicoccaceae; g__; s__	N	N

Table S8

OTU ID	Taxonomy	Fast Digester	All Digesters
752382	k__Bacteria; p__Bacteroidetes; c__Bacteroidia; o__Bacteroidales; f__; g__; s__	Y	Y
111350	k__Bacteria; p__Bacteroidetes; c__Bacteroidia; o__Bacteroidales; f__; g__; s__	Y	N
1144358	k__Bacteria; p__Bacteroidetes; c__Bacteroidia; o__Bacteroidales; f__; g__; s__	Y	N
4471301	k__Bacteria; p__Bacteroidetes; c__Bacteroidia; o__Bacteroidales; f__; g__; s__	Y	N
509055	k__Bacteria; p__Bacteroidetes; c__Bacteroidia; o__Bacteroidales; f__; g__; s__	N	N
562538	k__Bacteria; p__Bacteroidetes; c__Bacteroidia; o__Bacteroidales; f__; g__; s__	N	N
681365	k__Bacteria; p__Bacteroidetes; c__Bacteroidia; o__Bacteroidales; f__; g__; s__	N	N
787023	k__Bacteria; p__Bacteroidetes; c__Bacteroidia; o__Bacteroidales; f__; g__; s__	N	N
4468051	k__Bacteria; p__Bacteroidetes; c__Bacteroidia; o__Bacteroidales; f__Bacteroidaceae; g__; s__	Y	N
549570	k__Bacteria; p__Bacteroidetes; c__Bacteroidia; o__Bacteroidales; f__SB-1; g__; s__	Y	Y
550996	k__Bacteria; p__Firmicutes; c__Clostridia; o__Clostridiales; f__Lachnospiraceae; g__Coprococcus; s__	Y	N
3273469	k__Bacteria; p__Firmicutes; c__Clostridia; o__Clostridiales; f__Ruminococcaceae; g__; s__	Y	Y
562603	k__Bacteria; p__Proteobacteria; c__Deltaproteobacteria; o__Syntrophobacterales; f__Syntrophaceae; g__Syntrophus; s__	Y	Y
736489	k__Bacteria; p__Proteobacteria; c__Deltaproteobacteria; o__Syntrophobacterales; f__Syntrophaceae; g__Syntrophus; s__	Y	Y
279151	k__Bacteria; p__Tenericutes; c__Mollicutes; o__RF39; f__; g__; s__	Y	Y
111131	k__Bacteria; p__WWE1; c__[Cloacamonae]; o__[Cloacamonales]; f__[Cloacamonaceae]; g__Candidatus Cloacamonas; s__	Y	Y
534698	k__Bacteria; p__WWE1; c__[Cloacamonae]; o__[Cloacamonales]; f__[Cloacamonaceae]; g__W5; s__	N	N

Table S9

Day	Period	Number of Samples Processed Per Anaerobic digester			
		**Low A**	**Low B**	**Fast**	**Slow**
0	Startup	2	1	2	2
5	Startup	1	1	1	1
27	Startup	1	1	1	1
52	Startup	1	1	1	1
77	Startup	1	1	1	1
97	Startup	1	1	1	1
119	P1	1	1	1	1
133	P1	1	1	1	1
147	P1	1	1	1	1
175	P1	1	1	1	1
189	P1	1	1	1	1
201	P1	1	1	1	1
203	P2	1	1	1	1
209	P2	1	1	1	1
215	P2	1	1	1	1
217	P2	1	1	1	1
223	P2	1	1	1	1
231	P2	1	1	1	1
237	P2	1	1	1	1
243	P2	1	1	1	1
249	P2	1	1	1	1
255	P2	1	1	1	1
257	P2	1	1	1	1
265	P3	1	1	1	1
273	P3	1	1	1	1
281	P3	1	1	1	1
287	P3	1	1	1	1
291	P3	0	0	1	1
297	P3	1	1	1	1
299	P3	0	0	1	1
301	P3	1	1	1	1
307	P4	1	1	1	1
315	P4	1	1	0	1
321	P4	0	0	0	1
329	P4	1	1	0	1
333	P4	1	1	0	1
339	P4	0	0	0	1
343	P4	1	1	0	1
349	P4	1	1	1	1
355	P4	0	1	1	0

356	P4	0	1	1	0
357	P4	1	0	0	1
363	P4	1	0	0	1
369	P4	1	0	0	1
381	P4	1	0	0	1
383	P4	1	0	0	1
